# Supplementary figures and images for: Association between gestational weight gain and severe adverse birth outcomes in Washington State, US: A population-based retrospective cohort study, 2004–2013
Source: PLoS Med. 2019 Dec 30;16(12):e1003009. doi: 10.1371/journal.pmed.1003009 (PMC6936783; doi:10.1371/journal.pmed.1003009)

**S1 Form**. Washington State Birth Filing Form


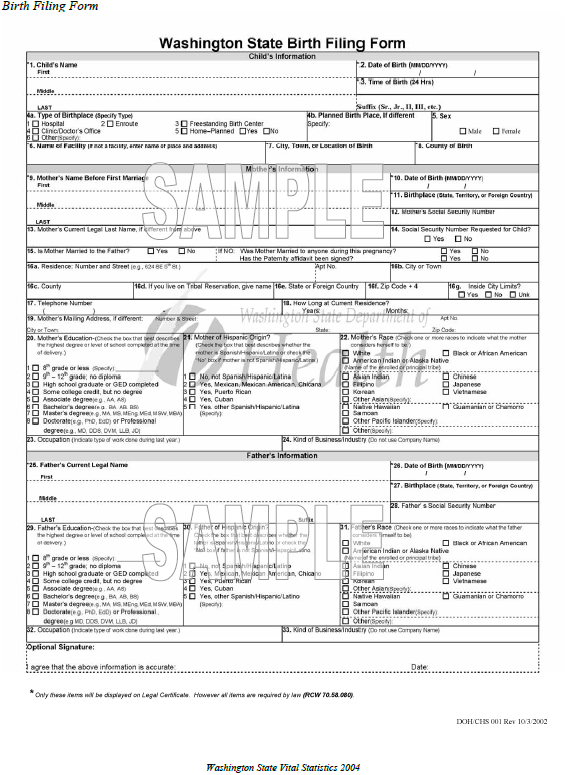


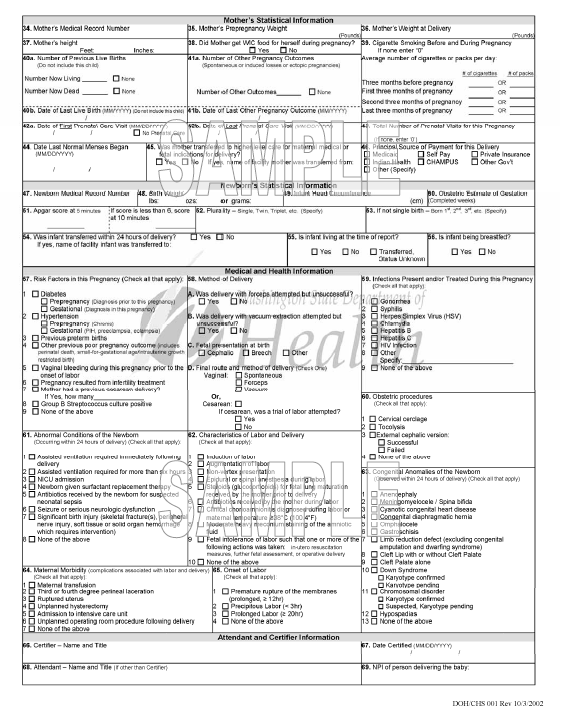


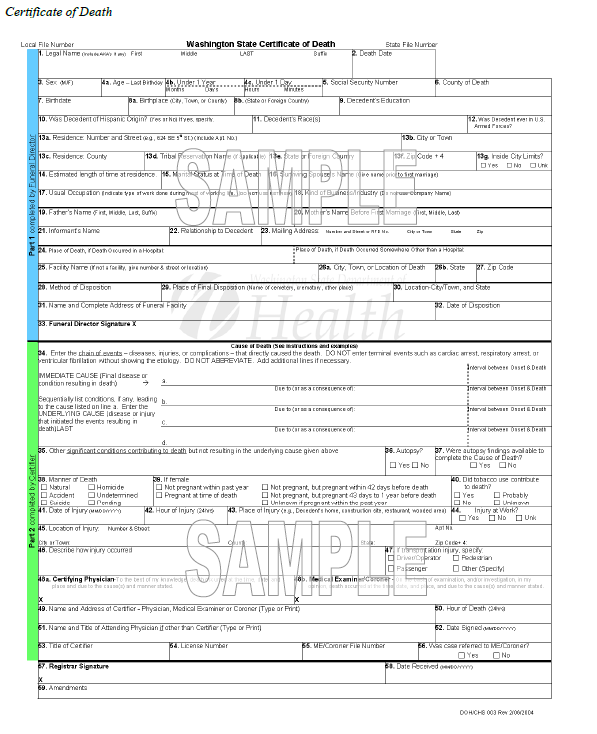

Supplement: S1 Form — (DOCX) [file pmed.1003009.s001.docx]
