# Supplementary material for: Association between gestational weight gain and severe adverse birth outcomes in Washington State, US: A population-based retrospective cohort study, 2004–2013
Source: PLoS Med. 2019 Dec 30;16(12):e1003009. doi: 10.1371/journal.pmed.1003009 (PMC6936783; doi:10.1371/journal.pmed.1003009)
Supplement: S1 Table — (DOCX) [file pmed.1003009.s003.docx]

**S1 Table.** Severe Maternal Morbidity definitions

| **Severe Maternal Morbidity** | **Conditions** |
| --- | --- |
| Antepartum hemorrhage with transfusion | Hemorrhage from placenta previa, premature separation of placenta or placental abruption, antepartum hemorrhage associated with coagulation defects, other or unspecified antepartum hemorrhage |
| Respiratory morbidity | Obstetric pulmonary embolism (including amniotic fluid embolism), pulmonary collapse, acute pulmonary edema, shock (lung), adult respiratory distress syndrome, acute cor pulmonale, pulmonary embolism (septic, iatrogenic, infection, other), respiratory arrest (unknown causes) |
| Thromboembolism or deep venous thrombosis | Arterial embolism and thrombosis of abdominal aorta, embolism or thrombosis of thoracic aorta, deep venous thrombosis, deep phlebothrombosis antepartum or postpartum, cerebral venous thrombosis including pulmonary thrombosis with clot (venous thromboembolism^a^) |
| Cerebrovascular or central nervous system morbidity | Subarachnoid hemorrhage, intracerebral hemorrhage, intracranial hemorrhage (including non-traumatic extradural hemorrhage, subdural hemorrhage, unspecified intracranial hemorrhage), occlusion or stenosis of pre-cerebral arteries, occlusion or stenosis of cerebral arteries, cerebral seizure, apoplexy, hypertensive encephalopathy, non-pyogenic thrombosis of intracranial venous system, hemiplegia, hemiparesis, other paralytic syndromes, encephalopathy, cerebrovascular disorders in puerperium |
| Cardiac morbidity | Malignant essential hypertension (with and without kidney involvement), malignant hypertension (with and without kidney involvement), acute myocardial infarction, aneurism of pulmonary artery, hemopericardium, constrictive pericarditis, cardiac tamponade, atrial fibrillation or flutter, ventricular fibrillation or flutter, cardiac arrest, heart failure, aortic aneurism and dissection, peripartum cardiomyopathy |
| Eclampsia | Eclampsia |
| Postpartum hemorrhage with transfusion | Postpartum hemorrhage (including PPH with coagulation defects) |
| Sepsis | Septicemia, septicemia during labor, major puerperal infection, septic shock, systemic inflammatory response syndrome (SIRS) |
| Acute renal failure | Acute and subacute renal failure, renal failure, unspecified other renal failure (after labor and delivery) |
| Obstetric shock | Obstetric shock |
| DIC | Disseminated intravascular coagulation |
| Uterine rupture | Rupture of uterus before onset of labor or during labor |
| Complication of anesthesia and obstetric interventions | Pulmonary complications, cardiac complications, CNS complications, other complications of surgery (cardiac arrest or failure after caesarean delivery), other anaphylactic shock, shock due to anesthesia, CNS complications (anoxic brain damage, cerebral hypoxia), iatrogenic cerebrovascular infarction (postoperative stroke), cardiac complications (during procedure), postoperative shock, air embolism (after infusion, perfusion, or transfusion) |
| Potentially lifesaving interventions | Conversion of cardiac rhythm, operations on heart (valves, septa, vessels), other operations on heart, temporary tracheostomy, other operations on vessels (e.g., shunts, stents, sutures), non-invasive mechanical ventilation, non-operative intubation, other continuous invasive mechanical ventilation, transfusion (any blood products), subtotal or total abdominal hysterectomy, other and unspecified hysterectomy, obstetric tamponade of uterus and vagina, invasive hemodynamic monitoring |
| Other | Acute and subacute liver failure, Budd-Chiari syndrome |
