# Supplementary material for: Association between gestational weight gain and severe adverse birth outcomes in Washington State, US: A population-based retrospective cohort study, 2004–2013
Source: PLoS Med. 2019 Dec 30;16(12):e1003009. doi: 10.1371/journal.pmed.1003009 (PMC6936783; doi:10.1371/journal.pmed.1003009)
Supplement: S2 Table — (DOCX) [file pmed.1003009.s004.docx]

**S2 Table**. Rates of maternal mortality and severe morbidity (rates per 10,000 births) by pre-pregnancy Body-Mass-Index and gestational weight gain, singleton births, Washington State, 2004-2013

|  | **Pre-pregnancy**  **underweight** | | | **Pre-pregnancy**  **normal BMI** | | | **Pre-pregnancy**  **overweight** | | | **Pre-pregnancy**  **obese** | | |
| --- | --- | --- | --- | --- | --- | --- | --- | --- | --- | --- | --- | --- |
|  | **Optimal weight gain** | **Low weight gain** | **Excess weight gain** | **Optimal weight**  **gain** | **Low weight**  **gain** | **Excess weight gain** | **Optimal weight**  **gain** | **Low weight**  **gain** | **Excess weight gain** | **Optimal weight**  **gain** | **Low weight**  **gain** | **Excess weight gain** |
| APH with transfusion | 9  (8.79) | 12  (8.43) | 7  (11.73) | 69  (5.20) | 64  (8.73) | 82  (5.79) | 41  (8.96) | 38  (14.46) | 78  (6.8) | 26  (6.64) | 37  (9.89) | 38  (4.25) |
| Respiratory morbidity | 7  (6.84) | 5  (7.68) | 14 (23.46) | 97  (7.32) | 68  (9.28) | 157 (11.08) | 38  (8.31) | 37  (14.08) | 110  (9.6) | 41 (10.47) | 51  (13.63) | 144  (16.12) |
| Obstetric embolism* | 1  (0.98) | 0 (0) | 2  (3.35) | 23  (1.73) | 10  (1.36) | 24  (1.69) | 6  (1.31) | 4  (1.52) | 15  (1.31) | 9  (2.3) | 10  (2.67) | 20  (2.24) |
| Thromboembolism or DVT | 3  (2.93) | 1  (1.54) | 5  (8.38) | 60  (4.53) | 44  (6) | 51  (3.6) | 37  (8.09) | 15  (5.71) | 54  (4.71) | 23  (5.87) | 34  (9.08) | 66  (7.39) |
| Cerebrovascular or CNS morbidity | 6  (5.86) | 7  (10.75) | 8  (13.41) | 79  (5.96) | 63  (8.6) | 79  (5.58) | 47 (10.27) | 17  (6.47) | 76 (6.63) | 32  (8.17) | 45  (12.02) | 88  (9.85) |
| Cardiac morbidity | 6  (5.86) | 4  (6.14) | 2  (3.35) | 40  (3.02) | 31  (4.23) | 62  (4.38) | 17  (3.72) | 17  (6.47) | 65 (5.67) | 26  (6.64) | 23  (6.15) | 58  (6.49) |
| Eclampsia | 2  (1.95) | 3  (4.61) | 3  (5.03) | 37  (2.79) | 32  (4.37) | 56  (3.95) | 15  (3.28) | 15  (5.71) | 53 (4.62) | 26 (6.64) | 23  (6.15) | 50  (5.6) |
| Severe PPH with transfusion | 45  (43.96) | 25  (38.4) | 28 (46.92) | 467 (35.23) | 318 (43.39) | 565 (39.87) | 165 (36.07) | 125 (47.56) | 428 (37.34) | 147 (37.54) | 115 (30.73) | 326  (36.5) |
| Severe PPH with coagulation defects | 15  (14.65) | 6  (9.22) | 13 (21.79) | 120 (9.05) | 90 (12.28) | 144 (10.16) | 120 (9.05) | 90 (12.28) | 144 (10.16) | 120 (9.05) | 90  (12.28) | 144  (10.16) |
| Sepsis (any) | 34  (33.21) | 25 (38.4) | 32 (53.63) | 351 (26.48) | 221 (30.15) | 551 (38.89) | 126 (27.54) | 82 (31.2) | 427 (37.25) | 120 (30.64) | 128 (34.2) | 382  (42.77) |
| Puerperal Sepsis | 27  (26.37) | 22 (33.79) | 28 (46.92) | 299 (22.55) | 176 (24.01) | 472 (33.31) | 103 (22.52) | 67 (25.49) | 374 (32.63) | 94  (24) | 100 (26.72) | 333  (37.28) |
| Acute renal failure | 2  (1.95) | 2  (3.07) | 2  (3.35) | 8  (0.60) | 11  (1.5) | 19  (1.34) | 6  (1.31) | 6  (2.28) | 27  (2.36) | 7  (1.79) | 6  (1.6) | 29  (3.25) |
| Hepatic failure | 0  (0) | 0  (0) | 0  (0) | 1  (0.08) | 1  (0.14) | 4  (0.28) | 1  (0.22) | 0  (0) | 1  (0.09) | 0  (0) | 1  (0.27) | 3  (0.34) |
| Obstetric shock | 2  (1.95) | 3  (4.61) | 0  (0) | 22  (1.66) | 10  (1.36) | 24  (1.69) | 5  (1.09) | 8  (3.04) | 10  (0.87) | 7  (1.79) | 6  (1.6) | 16  (1.79) |
| DIC | 5  (4.88) | 0  (0) | 3  (5.03) | 34  (2.56) | 21  (2.87) | 28  (1.98) | 10  (2.19) | 7  (2.66) | 18  (1.57) | 9  (2.3) | 10  (2.67) | 12  (1.34) |
| Uterine rupture | 3  (2.93) | 1  (1.54) | 3  (5.03) | 56  (4.22) | 31  (4.23) | 55  (3.88) | 26  (5.68) | 20  (7.61) | 39  (3.4) | 22  (5.62) | 26  (6.95) | 39  (4.37) |
| Complications of anesthesia or obstetric interventions | 18  (17.58) | 8  (12.29) | 11 (18.43) | 194 (14.63) | 98 (13.37) | 232 (16.37) | 80 (17.49) | 43 (16.36) | 212 (18.49) | 82 (20.94) | 84  (22.44) | 194  (21.72) |
| Potentially lifesaving interventions | 84  (82.06) | 54 (82.95) | 59 (98.88) | 837 (63.14) | 591 (80.64) | 1050 (74.1) | 357 (78.04) | 261 (99.3) | 838  (73.1) | 306 (78.14) | 301 (80.43) | 711  (79.6) |
| Hysterectomy | 12  (11.72) | 1  (1.54) | 5  (8.38) | 78  (5.88) | 41  (5.59) | 75  (5.29) | 32  (7) | 21  (7.99) | 64  (5.58) | 24  (6.13) | 38  (10.15) | 56  (6.27) |
| Blood or blood products transfusion | 66  (64.47) | 49 (75.27) | 50 (83.79) | 683 (51.52) | 512 (69.86) | 864 (60.98) | 298 (65.14) | 224 (85.22) | 695 (60.63) | 235 (60.01) | 239 (63.86) | 537  (60.12) |
| Respiratory (assisted ventilation) | 2  (1.95) | 2  (3.07) | 5  (8.38) | 18  (1.36) | 25  (3.41) | 48  (3.39) | 16  (3.5) | 12  (4.57) | 40  (3.49) | 23  (5.87) | 21  (5.61) | 53  (5.93) |
| ICU admission | 10  (9.77) | 7  (10.75) | 9  (15.08) | 87  (6.56) | 56  (7.64) | 108 (7.62) | 29  (6.34) | 31 (11.79) | 98  (8.55) | 33  (8.43) | 47  (12.56) | 92  (10.3) |
| Death | 0  (0) | 1  (1.54) | 0  (0) | 1  (0.08) | 5  (0.68) | 7  (0.49) | 4  (0.87) | 0  (0) | 4  (0.35) | 1  (0.26) | 5  (1.34) | 3  (0.34) |
| Composite death/SMM | 158 (154.34) | 102 (156.68) | 116 (194.4) | 1660 (125.21) | 1133 (154.59) | 2179 (153.78) | 690 (150.83) | 499 (189.85) | 1792 (156.33) | 620 (158.32) | 655 (175.02) | 1616 (180.92) |

Abbreviations: APH, antepartum hemorrhage; DIC, disseminated Intravascular Coagulation; PPH, postpartum hemorrhage; ICU -Intensive Care Unit; SMM,severe maternal morbidity.

*Includes amniotic fluid, air, and blood clot embolism.
