# Supplementary material for: Association between gestational weight gain and severe adverse birth outcomes in Washington State, US: A population-based retrospective cohort study, 2004–2013
Source: PLoS Med. 2019 Dec 30;16(12):e1003009. doi: 10.1371/journal.pmed.1003009 (PMC6936783; doi:10.1371/journal.pmed.1003009)
Supplement: S3 Table — (DOCX) [file pmed.1003009.s005.docx]

**S3 Table**. Adjusted Odds Ratios (AOR) for SMM components by gestational weight gain and pre-pregnancy Body Mass Index ^a^, singleton births, Washington State, 2004-2013 (AOR relative to optimal weight gain in each pre-pregnancy Body Mass Index category).

| **Mortality/SMM** | **Pre-pregnancy**  **Underweight** | | | **Pre-pregnancy**  **Normal BMI** | | | **Pre-pregnancy**  **Overweight** | | | **Pre-pregnancy**  **Obese** | | |
| --- | --- | --- | --- | --- | --- | --- | --- | --- | --- | --- | --- | --- |
|  | **L-GWG**  AOR*  (95% CI) | **O-GWG**  (Ref) | **E-GWG**  AOR*  (95% CI) | **L-GWG**  AOR*  (95% CI) | **O-GWG**  (Ref) | **E-GWG**  AOR*  (95% CI) | **L-GWG**  AOR*  (95% CI) | **O-GWG**  (Ref) | **E-GWG**  AOR*  (95% CI) | **L-GWG**  AOR*  (95% CI) | **O-GWG**  (Ref) | **E-GWG**  AOR*  (95% CI) |
| Antepartum haemorrhage with transfusion | 2.03  (0.85-4.85) | 1 | 1.13  (0.41- 3.12) | 1.41  (0.99-2.00) | 1 | 1.15  (0.83-1.60) | 1.32  (0.83-2.11) | 1 | 0.89  (0.60-1.31) | 1.30  (0.77-2.20) | 1 | 0.69  (0.41-1.15) |
| Respiratory morbidity including embolism | 1.11  (0.35-3.52) | 1 | **3.40**  **(1.35- 8.60)** | 1.03  (0.74-1.43) | 1 | 1.47  (0.11-1.91) | 1.54  (0.96-2.45) | 1 | 1.21  (0.82-1.78) | 1.32  (0.86-2.01) | 1 | **1.56**  **(1.09-2.23)** |
| Thromboembolism or DVT | 0.64  (0.06-7.14) | 1 | 3.20  (0.57-1.80) | 1.38  (0.93-2.06) | 1 | 0.74  (0.51-1.09) | 0.68  (0.37-1.25) | 1 | **0.54**  **(0.35-0.82)** | 1.59  (0.94-2.70) | 1 | 1.19  (0.74-1.93) |
| Cerebrovascular or CNS morbidity | 2.01  (0.64- 6.39) | 1 | 2.35  (0.73-7.51) | **1.47**  **(1.05-2.06)** | 1 | 0.86  (0.62-1.18) | 0.61  (0.35-1.09) | 1 | **0.58**  **(0.40-0.85)** | 1.50  (0.96-2.37) | 1 | 1.13  (0.75-1.70) |
| Cardiac morbidity | 0.79  (0.20-3.29) | 1 | 0.53  (0.10-2.67) | 1.19  (0.07-1.94) | 1 | 1.40  (0.94-2.10) | 1.53  (0.76-3.07) | 1 | 1.45  (0.84-2.51) | 0.81  (4.49-1.46) | 1 | 1.05  (0.65-1.68) |
| Eclampsia | 1.69  (0.24-1.21) | 1 | 2.25  (0.37-1.37) | 1.58  (0.97-2.56) | 1 | 1.25  (0.81-1.92) | 1.58  (0.76-3.29) | 1 | 1.29  (0.72-2.31) | 1.01  (0.57-1.78) | 1 | 0.81  (0.50-1.32) |
| Severe postpartum haemorrhage with transfusion | 0.85  (0.52-1.40) | 1 | 1.11  (0.69-1.80) | 1.12  (0.96-1.29) | 1 | **1.14**  **(1.01-1.29)** | 1.26  (1.00-1.60) | 1 | 1.10  (0.91-1.32) | 0.82  (0.64-1.05) | 1 | 1.01  (0.83-1.23) |
| Sepsis | 1.21  (0.70-2.07) | 1 | **1.82**  **(1.10-3.02)** | 1.03  (0.87-1.22) | 1 | **1.37**  **(1.19-1.57)** | 0.98  (0.74-1.32) | 1 | **1.31**  **(1.07-1.61)** | 1.10  (0.85-1.43) | 1 | **1.27**  **(1.03-1.57)** |
| Puerperal sepsis | 1.37  (0.75-2.48) | 1 | **2.08**  **(1.19-3.64)** | 0.97  (0.80-1.17) | 1 | **1.37**  **(1.18-1.59)** | 0.98  (0.71-1.35) | 1 | **1.39**  **(1.11-1.74)** | 1.08  (0.80-1.44) | 1 | **1.39**  **(1.09-1.75)** |
| Acute renal failure | 1.57  (0.22-11.21) | 1 | 2.44  (0.34-17.51) | 2.39  (0.95-5.99) | 1 | 2.06  (0.89-4.76) | 1.74  (0.56-5.42) | 1 | 1.73  (0.71-4.25) | 1.05  (0.34-3.28) | 1 | 2.27  (0.94-5.50) |
| Hepatic failure | - | - | - | 1.51  (0.09-24.55) | 1 | 4.02  (0.44-36.79) | - | 1 | 0.34  (0.02-5.90) | - | 1 | - |
| Obstetric shock | 2.24  (0.37-13.50) | 1 | - | 0.79  (0.36-1.73) | 1 | 1.05  (0.57-1.92) | 2.39  (0.76-7.50) | 1 | 0.95  (0.32-2.81) | 0.91  (0.31-2.72) | 1 | 1.07  (0.44-2.63) |
| DIC | - | 1 | 1.62  (0.38-6.88) | 0.84  (0.46-1.51) | 1 | 0.83  (0.49-1.40) | 1.03  (0.37-2.85) | 1 | 0.82  (0.38-1.79) | 1.18  (0.45-3.05) | 1 | 0.65  (0.26-1.63) |
| Uterine rupture | 0.45  (4.63-4.43) | 1 | 1.78  (0.34-9.58) | 0.87  (0.55-1.38) | 1 | 1.02  (0.70-1.50) | 1.25  (0.69-2.26) | 1 | 0.69  (0.41-1.14) | 1.16  (0.65-2.06) | 1 | 0.88  (0.52-1.50) |
| Complications of anesthesia or obstetric interventions | 0.67  (0.29-1.54) | 1 | 0.95  (0.44-2.03) | 0.90  (0.70-1.15) | 1 | 1.16  (0.96-1.41) | 0.91  (0.62-1.33) | 1 | 1.07  (0.82-1.39) | 1.06  (0.78-1.45) | 1 | 1.05  (0.80-1.36) |
| Potentially lifesaving interventions | 0.92  (0.65-1.31) | 1 | 1.23  (0.88-1.73) | **1.13**  **(1.01-1.26)** | 1 | **1.18**  **(1.08-1.30)** | 1.05  (0.60-1.85) | 1 | 0.96  (0.63-1.48) | 0.99  (0.84-1.16) | 1 | 1.07  (0.93-1.22) |
| Hysterectomy | **0.12**  **(0.02-0.94)** | 1 | 0.90  (0.31-2.60) | 0.86  (0.58-1.27) | 1 | 1.04  (0.75-1.45) | 1.05  (0.60-1.85) | 1 | 0.96  (0.63-1.48) | 1.60  (0.95-2.70) | 1 | 1.21  (0.74-1.98) |
| Blood or blood products transfusion | 1.07  (0.74-1.57) | 1 | 1.31  (0.90-1.92) | **1.19**  **(1.06-1.35)** | 1 | **1.17**  **(1.06-1.30)** | 1.19  (1.00-1.43) | 1 | 0.99  (0.86-1.13) | 1.02  (0.85-1.23) | 1 | 1.05  (0.90-1.23) |
| Respiratory (assisted ventilation) | 0.85  (0.08-9.41) | 1 | 3.32  (0.63-1.74) | 1.45  (0.75-2.81) | 1 | **2.54**  **(1.46-4.41)** | 1.10  (0.49-2.45) | 1 | 1.24  (0.67-2.29) | 0.96  (0.53-1.73) | 1 | 1.01  (0.61-1.65) |
| ICU admission | 0.89  (0.32-2.47) | 1 | 1.73  (0.69-4.32) | 1.03  (0.73-1.45) | 1 | 1.13  (0.84-1.51) | **1.99**  **(1.19-3.34)** | 1 | **1.54**  **(1.00-2.37)** | 1.48  (0.94-2.31) | 1 | 1.23  (0.82-1.84) |
| Composite: maternal death/severe morbidity | 0.96  (0.74-1.24) | 1 | 1.28  (1.00-1.63) | **1.12**  **(1.04-1.21)** | 1 | **1.20**  **(1.12-1.28)** | **1.17**  **(1.04-1.32)** | 1 | 1.07  (0.98-1.18) | 1.07  (0.95-1. 02) | 1 | **1.12**  **(1.01-1.23)** |

Abbreviation: BMI, body mass index; O-GWG, optimal gestational weight gain; L-GWG low gestational weight gain; E-GWG, excess gestational weight gain; SMM Severe maternal morbidity; ICU, intensive care unit; DVT, deep vein thrombosis; DIC disseminated intravascular coagulation.

AOR adjusted for maternal age ( <25yrs, 25-35yrs, ≥35yrs), maternal education (high school graduation or higher vs less than high school graduation), marital status (single, widowed, or separated vs married or common law), race/ethnicity (Hispanic, African American, Native American, and other vs non-Hispanic white), parity (nulliparous, parity ≥4 vs parity 1-3), assisted conception (no vs yes), smoking during pregnancy (no vs yes), type of health insurance (Medicaid, private vs other), year of birth, and fetal sex (female vs male).

^±^2-sided p-values were calculated using multivariable logistic regressions Wald Chi-square test.
