# Supplementary material for: Association between gestational weight gain and severe adverse birth outcomes in Washington State, US: A population-based retrospective cohort study, 2004–2013
Source: PLoS Med. 2019 Dec 30;16(12):e1003009. doi: 10.1371/journal.pmed.1003009 (PMC6936783; doi:10.1371/journal.pmed.1003009)
Supplement: S4 Table — (DOCX) [file pmed.1003009.s006.docx]

**S4 Table**. Adjusted Odds Ratios (AOR) for stillbirth, neonatal death and severe neonatal morbidity by gestational weight gain and pre-pregnancy Body Mass Index, singleton births, Washington State, 2004-2013 (AOR relative to optimal weight gain in each pre-pregnancy Body Mass Index category).

| **Pre-pregnancy BMI** | **Underweight** | | **Normal BMI** | | **Overweight** | | **Obese** | |
| --- | --- | --- | --- | --- | --- | --- | --- | --- |
| **Outcomes** | **Low weight gain**  AOR  (95% CI)  *P*-value^±^ | **Excess weight gain**  AOR  (95% CI)  *P*-value^±^ | **Low weight gain**  AOR  (95% CI)  *P*-value^±^ | **Excess weight gain**  AOR  (95% CI)  *P*-value^±^ | **Low weight gain**  AOR  (95% CI)  *P*-value^±^ | **Excess weight gain**  AOR  (95% CI)  *P*-value^±^ | **Low weight gain**  AOR  (95% CI)  *P*-value^±^ | **Excess weight gain**  AOR  (95% CI)  *P*-value^±^ |
| Stillbirth | 1.58  (0.83-3.00)  *0.162* | 0.76  (0.35-1.66)  *0.490* | **2.17**  **(1.81-2.61)**  *<0.001* | **1.44**  **(1.20-1.71)**  *<0.001* | **3.21**  **(2.50-4.11)**  *<0.001* | 0.86  (0.67-1.09)  *0.191* | **2.59**  **(2.06-3.26)**  *<0.001* | 0.86  (0.68-1.09)  *0.269* |
| Neonatal death | **2.79**  **(1.32-5.88)**  *0.007* | **2.21**  **(1.00-4.86)**  *0.049* | **2.07**  **(1.68-2.55)**  *<0.001* | **1.38**  **(1.13-1.69)**  *0.002* | **1.73**  **(1.27-2.36)**  *0.001* | 0.89  (0.68-1.16)  *0.307* | **1.44**  **(1.11-1.87)**  *0.007* | **0.77**  **(0.60-0.99)**  *0.05* |
| Severe neonatal morbidity | **1.88**  **(1.41-2.05)**  *<0.001* | **1.52**  **(1.13-2.05)**  *0.006* | **1.16**  **(1.06-1.28)**  *<0.001* | **1.37**  **(1.27-1.48)**  *<0.001* | **1.28**  **(1.11-1.47)**  *<0.001* | 1.04  (0.93-1.16)  *0.563* | **1.33**  **(1.18-1.51)**  *<0.001* | **1.16**  **(1.04-1.29)**  *0.006* |
| Perinatal death or severe neonatal morbidity | **1.92**  **(1.50-2.46)**  *<0.001* | **1.49**  **(1.15-1.95)**  *0.003* | **1.41**  **(1.31-1.53)**  *<0.001* | **1.38**  **(1.29-1.85)**  *<0.001* | **1.65**  **(1.47-1.84)**  *<0.001* | 0.99  (0.90-1.08)  *0.808* | **1.55**  **(1.40-1.72)**  *<0.001* | 1.05  (0.96-1.15)  *0.203* |
| Small-for-gestational age | **1.90**  **(1.74-2.07)**  *<0.001* | **0.56**  **(0.49-0.62)**  *<0.001* | **1.72**  **(1.67-1.78)**  *<0.001* | **0.65**  **(0.63-0.67)**  *<0.001* | **1.35**  **(1.28-1.43)**  *<0.001* | **0.61**  **(0.58-0.64)**  *<0.001* | **1.19**  **(1.12-1.26)**  *<0.001* | **0.75**  **(0.71-0.79)**  *<0.001* |
| Large-for-gestational age | **0.39**  **(0.30-0.50)**  *<0.001* | **2.42**  **(2.07-2.83)**  *<0.001* | **0.55**  **(0.53-0.58)**  *<0.001* | **2.13**  **(2.06-2.19)**  *<0.001* | **0.70**  **(0.66-0.75)**  *<0.001* | **2.00**  **(1.93-2.09)**  *<0.001* | **0.80**  **(0.76-0.83)**  *<0.001* | **1.61**  **(1.56-1.67)**  *<0.001* |

AOR adjusted for maternal age ( <25yrs, 25-35yrs, ≥35yrs), maternal education (high school graduation or higher vs less than high school graduation), marital status (single, widowed, or separated vs married or common law), race/ethnicity (Hispanic, African American, Native American, and other vs non-Hispanic white), parity (nulliparous, parity ≥4 vs parity 1-3), assisted conception (no vs yes), smoking during pregnancy (no vs yes), type of health insurance (Medicaid, private vs other), year of birth, fetal sex (female vs male), and congenital anomalies.

^±^2-sided p-values were calculated using multivariable logistic regressions Wald Chi-square test.
