# Supplementary material for: Association between gestational weight gain and severe adverse birth outcomes in Washington State, US: A population-based retrospective cohort study, 2004–2013
Source: PLoS Med. 2019 Dec 30;16(12):e1003009. doi: 10.1371/journal.pmed.1003009 (PMC6936783; doi:10.1371/journal.pmed.1003009)
Supplement: S5 Table — (DOCX) [file pmed.1003009.s007.docx]

**S5 Table**. Rates of maternal death and severe morbidity by gestational weight gain and pre-pregnancy **obesity class**, singleton births, Washington State, 2004-2013; No (rates per 10,000)

|  | **Pre-pregnancy**  **Obese class 1** | | | **Pre-pregnancy**  **Obese class 2** | | | **Pre-pregnancy**  **Obese class 3** | | |
| --- | --- | --- | --- | --- | --- | --- | --- | --- | --- |
|  | **O-GWG** | **L-GWG** | **E-GWG** | **O-GWG** | **L-GWG** | **E-GWG** | **O-GWG** | **L-GWG** | **E-GWG** |
| APH with transfusion | 19 (9.07) | 19 (11.45) | 26 (4.62) | 7 (6.33) | 13 (11.52) | 8 (3.64) | 0 (0) | 5 (5.24) | 4 (3.62) |
| Respiratory | 18 (8.6) | 20 (12.05) | 70 (12.43) | 10 (9.04) | 13 (11.52) | 39 (17.74) | 13 (18.16) | 18 (18.86) | 35 (31.71) |
| Obstetric embolism | 6 (2.87) | 3 (1.81) | 11 (1.95) | 1 (0.9) | 2 (1.77) | 6 (2.73) | 2 (2.79) | 5 (5.24) | 3 (2.72) |
| Amniotic fluid embolism | 3 (1.43) | 3 (1.81) | 4 (0.71) | 1 (0.9) | 0 (0) | 1 (0.45) | 0 (0) | 0 (0) | 0 (0) |
| Blood clot embolism (VTE) | 3 (1.43) | 0 (0) | 5 (0.89) | 0 (0) | 2 (1.77) | 5 (2.27) | 2 (2.79) | 5 (5.24) | 3 (2.72) |
| Thromboembolism or DVT | 12 (5.73) | 12 (7.23) | 43 (7.64) | 6 (5.42) | 10 (8.86) | 14 (6.37) | 5 (6.98) | 12 (12.57) | 9 (8.16) |
| Cerebrovascular or CNS morbidity | 18 (8.6) | 15 (9.04) | 60 (10.66) | 7 (6.33) | 13 (11.52) | 17 (7.73) | 7 (9.78) | 17 (17.81) | 11 (9.97) |
| Cerebral venous thrombosis | 2 (0.96) | 3 (1.81) | 12 (2.13) | 0 (0) | 2 (1.77) | 0 (0) | 0 (0) | 0 (0) | 2 (1.81) |
| Cerebrovascular disorders in the puerperium | 3 (1.43) | 0 (0) | 6 (1.07) | 0 (0) | 0 (0) | 1 (0.45) | 1 (1.4) | 1 (1.05) | 1 (0.91) |
| Cardiac morbidity | 10 (4.78) | 8 (4.82) | 29 (5.15) | 6 (5.42) | 5 (4.43) | 14 (6.37) | 10 (13.97) | 10 (10.48) | 15 (13.59) |
| Cardiac arrest or failure or MI | 6 (2.87) | 5 (3.01) | 11 (1.95) | 3 (2.71) | 4 (3.54) | 6 (2.73) | 3 (2.71) | 4 (3.54) | 6 (2.73) |
| Peripartum cardiomyopathy | 2 (0.96) | 0 (0) | 7 (1.24) | 0 (0) | 0 (0) | 4 (1.82) | 4 (5.59) | 0 (0) | 3 (2.72) |
| Eclampsia | 8 (3.82) | 10 (6.03) | 25 (4.44) | 10 (9.04) | 9 (7.97) | 17 (7.73) | 8 (11.17) | 4 (4.19) | 8 (7.25) |
| severe PPH with transfusion | 82 (39.16) | 55 (33.15) | 211 (37.47) | 43 (38.88) | 40 (35.44) | 77 (35.03) | 22 (30.73) | 20 (20.95) | 38 (34.43) |
| severe PPH With coagulation defects | 20 (9.55) | 19 (11.45) | 59 (10.48) | 9 (8.14) | 6 (5.32) | 11 (5) | 9 (8.14) | 6 (5.32) | 11 (5) |
| Sepsis | 63 (30.09) | 57 (34.35) | 216 (38.36) | 34 (30.74) | 37 (32.78) | 114 (51.86) | 23 (32.12) | 34 (35.62) | 52 (47.12) |
| Puerperal Sepsis | 51 (24.36) | 46 (27.72) | 183 (32.5) | 26 (23.51) | 31 (27.46) | 103 (46.86) | 17 (23.74) | 23 (24.1) | 47 (42.59) |
| Acute renal failure | 1 (0.48) | 3 (1.81) | 17 (3.02) | 4 (3.62) | 2 (1.77) | 7 (3.18) | 2 (2.79) | 1 (1.05) | 5 (4.53) |
| Hepatic failure | 0 (0) | 0 (0) | 2 (0.36) | 0 (0) | 0 (0) | 0 (0) | 0 (0) | 1 (1.05) | 1 (0.91) |
| Obstetric shock | 2 (0.96) | 2 (1.21) | 8 (1.42) | 4 (3.62) | 0 (0) | 6 (2.73) | 1 (1.4) | 4 (4.19) | 2 (1.81) |
| DIC | 4 (1.91) | 9 (5.42) | 8 (1.42) | 5 (4.52) | 0 (0) | 2 (0.91) | 0 (0) | 1 (1.05) | 2 (1.81) |
| Uterine rupture | 16 (7.64) | 12 (7.23) | 26 (4.62) | 4 (3.62) | 7 (6.2) | 7 (3.18) | 2 (2.79) | 7 (7.33) | 6 (5.44) |
| Complications of anesthesia or obstetric interventions | 39 (18.62) | 32 (19.29) | 120 (21.31) | 29 (26.22) | 28 (24.81) | 41 (18.65) | 14 (19.55) | 24 (25.14) | 33 (29.9) |
| Potentially lifesaving interventions | 169 (80.71) | 140 (84.38) | 427 (75.84) | 79 (71.43) | 89 (78.84) | 176 (80.07) | 58 (81.01) | 72 (75.43) | 108 (97.86) |
| Hysterectomy | 11 (5.25) | 16 (9.64) | 30 (5.33) | 8 (7.23) | 13 (11.52) | 18 (8.19) | 5 (6.98) | 9 (9.43) | 8 (7.25) |
| Blood or blood products transfusion | 135 (64.47) | 116 (69.91) | 327 (58.08) | 58 (52.44) | 76 (67.33) | 133 (60.51) | 42 (58.66) | 47 (49.24) | 77 (69.77) |
| Respiratory (assisted ventilation) | 12 (5.73) | 8 (4.82) | 27 (4.8) | 5 (4.52) | 5 (4.43) | 11 (5) | 6 (8.38) | 8 (8.38) | 15 (13.59) |
| ICU admission | 12 (5.73) | 20 (12.05) | 49 (8.7) | 8 (7.23) | 8 (7.09) | 27 (12.28) | 13 (18.16) | 19 (19.91) | 16 (14.5) |
| Death | 0 (0) | 1 (0.6) | 3 (0.53) | 1 (0.9) | 2 (1.77) | 0 (0) | 0 (0) | 2 (2.1) | 0 (0) |
| Composite SMM | 328 (156.64) | 288 (173.58) | 952 (169.08) | 170 (153.71) | 194 (171.86) | 414 (188.34) | 122 (170.39) | 173 (181.25) | 250 (226.53) |

Abbreviation: O-GWG, optimal gestational weight gain; L-GWG low gestational weight gain; E-GWG, excess gestational weight gain; APH, antepartum hemorrhage; VTE, venous thromboembolism; DVT, Deep vein thrombosis; MI, myocardial infarction; PPH, postpartum hemorrhage; DIC, disseminated intravascular coagulation; ICU, Intensive care unit; SMM, severe maternal morbidity.
