# Supplementary material for: Association between gestational weight gain and severe adverse birth outcomes in Washington State, US: A population-based retrospective cohort study, 2004–2013
Source: PLoS Med. 2019 Dec 30;16(12):e1003009. doi: 10.1371/journal.pmed.1003009 (PMC6936783; doi:10.1371/journal.pmed.1003009)
Supplement: S6 Table — (DOCX) [file pmed.1003009.s008.docx]

**S6 Table**. Adjusted Odds Ratios (AOR) for the components of SMM by gestational weight gain and pre-pregnancy **obesity class**, singleton births, Washington State, 2004-2013^a^ (AOR Relative to optimal weight gain in each pre-pregnancy obesity class)

| **SMM** | **Pre-pregnancy**  **Obese class 1** | | | **Pre-pregnancy**  **Obese class 2** | | | **Pre-pregnancy**  **Obese class 3** | | |
| --- | --- | --- | --- | --- | --- | --- | --- | --- | --- |
|  | **L-GWG**  AOR*  (95% CI) | **O-GWG**  (Ref) | **E-GWG**  AOR*  (95% CI) | **L-GWG**  AOR*  (95% CI) | **O-GWG**  (Ref) | **E-GWG**  AOR*  (95% CI) | **L-GWG**  AOR*  (95% CI) | **O-GWG**  (Ref) | **E-GWG**  AOR*  (95% CI) |
| APH with transfusion | 1.20  (0.62-2.34) | 1 | 5.56  (0.30-1.03) | 1.55  (0.61- 3.94) | 1 | 0.66  (0.24-1.83) | - | 1 | - |
| Respiratory morbidity | 1.36  (0.70-2.65) | 1 | 1.44  (0.84-2.45) | 1.36  (0.58- 3.20 ) | 1 | 2.03  (0.97-4.22) | 1.02  (0.51-2.09) | 1 | 1.63  (0.86-3.10) |
| Obstetric embolism | 0.64  (0.12-2.57) | 1 | 0.58  (0.20-1.65) | 0 | 1 | 0 | 1.93  (0.37- 9.96) | 1 | 1.02  (0.17-6.18) |
| Thromboembolism or DVT | 1.29  (0.58-2.88) | 1 | 1.15  (0.60-2.20) | 1.70  (0.62-4.69) | 1 | 1.24  (0. 48-3.26) | 1.86  (0.65-5.30) | 1 | 1.18  (0.39- 3.55) |
| Cerebrovascular or CNS morbidity | 1.06  (0.54-2.11) | 1 | 1.07  (0.63-1.83) | 1.86  (0.74-4.67) | 1 | 1.32  (0.55- 3.21) | 1.91  (0.79-4.64) | 1 | 0.98  (0.38-2.54) |
| Cardiac morbidity | 0.75  (0.27-2.06) | 1 | 1.13  (0.54-2.33) | 0.73  (0.20- 2.75) | 1 | 1.32  (0.47-3.76) | 0.75  (0.31-1.80) | 1 | 0.95  (0.42-2.13) |
| Eclampsia | 1.46  (0.58-3.71) | 1 | 9.63  (0.43-2.15) | 0.99  (0.39-2.47) | 1 | 0.86  (0. 38-1.94) | 0.43  (0.13-1.45) | 1 | 0.68  (0.24-1.90) |
| Severe PPH with transfusion | 0.86  (0.61-1.21) | 1 | 0.98  (0.75-1.27) | 0.90  (0.58-1.42) | 1 | 0.95  (0.65-1.40) | 0.64  ( 0.34- 1.18) | 1 | 1.10  ( 0.65-1.87) |
| severe PPH With coagulation defects | 1.21  (0.64-2.26) | 1 | 1.10  (0.65-1.84) | 0.82  (0.27- 2.45) | 1 | 0.74  (0.28-1.92) | 2.26  (0.23-21.80) | 1 | 6.56  (0.83-52.06) |
| Sepsis | 1.05  (0.73-1.52) | 1 | 1.11  (0.84-1.49) | 1.19  (0.73-1.93) | 1 | **1.62**  **(1.08-2.43)** | 1.03  (0.60-1.78) | 1 | 1.26  (0.77-2.07) |
| Puerperal Sepsis | 1.03  (0.69-1.55) | 1 | 1.13  (0.83-1.56) | 1.28  (0.74-2.21) | 1 | **1.87**  **(1.18-2.96)** | 0.90  (0.47-1.73) | 1 | 1.49  (0.85-2.61) |
| Acute renal failure | 3.86  (0.40-3.73) | 1 | 7.11  (0.93-5.42) | 0.64  (0.11-3.89) | 1 | 1.19  (0.30- 4.68) | 0.37  (0.03- 4.18) | 1 | 1.60  (0.30-8.57) |
| Obstetric shock | 1.59  (0.22-11.45) | 1 | 1.88  (0.39-9.01) | 0 | 1 | 0.67  (0.18-2.42) | 3.63  (0.37-35.31) | 1 | 1.40  (0.12-16.66) |
| DIC | 2.58  (0.78-8.61) | 1 | 0.70  (0.20-2.40) | 0 | 1 | 0.21  (0.04-1.19) | 0 | 1 | 0 |
| Uterine rupture | 0.86  (0.40-1.86) | 1 | 0.68  (0.40-1.28) | 1.67  (0.49-5.71) | 1 | 1.05  (0.31- 3.61) | 2.49  (0.51-12.07) | 1 | 2.26  (0.45-11.29) |
| Complications of anesthesia or obstetric interventions | 1.05  (0.65-1.70) | 1 | 1.17  (0.80-1.70) | 0.94  (0.56- 1.58) | 1 | 0.69  (0.43-1.11) | 1.20  (0.62- 2.33) | 1 | 1.44  (0.77-2.73) |
| Potentially lifesaving interventions | 1.02  (0.81-1.28) | 1 | 1.00  (0.83-1.20) | 1.03  (0.76-1.41) | 1 | 1.15  (0. 88-1.51) | 0. 88  (0.62-1.25) | 1 | 1.14  (0. 82-1.57) |
| Hysterectomy | 1.64  (0.75-3.59) | 1 | 1.14  (0.56-2.29) | 1.73  (0.69-4.35) | 1 | 1.48  (0.61-3.59) | 1.24  (0.41-3.73) | 1 | 0. 97  (0.30-3.08) |
| Blood or blood products transfusion | 1.06  (0.82-1.37) | 1 | 0.95  (0.78-1.17) | 1.21  (0.85- 1.71) | 1 | 1.18  (0. 86-1.62) | 0.77  (0.50- 1.18) | 1 | 1.12  (0.77-1.65) |
| Respiratory (assisted ventilation) | 0.85  (0.35-2.08) | 1 | 0.79  (0.40-1.58) | 0. 93  (0.27-3.23) | 1 | 1.04  (0.36-3.04) | 0. 97  (0.33-2.82) | 1 | 1.44  (0.55-3.79) |
| ICU admission | **2.12**  **(1.04-4.35)** | 1 | 1.50  (0.79-2.85) | 0. 99  (0.37-2.65) | 1 | 1.74  (0.79-3.86) | 1.03  (0.50-2.10) | 1 | 0.73  (0.35-1.55) |
| Composite SMM | 1.05  (0.90-1.25) | 1 | 1.07  (0.94-1.22) | 1.10  (0.88-1.36) | 1 | 1.20  (1.00-1.44) | 1.03  (0.81-1.30) | 1 | 1.23  (0.99-1.54) |

Abbreviations: O-GWG, optimal gestational weight gain; L-GWG low gestational weight gain; E-GWG, excess gestational weight gain; APH, antepartum hemorrhage; DVT, deep vein thrombosis; CNS, central nervous system; PPH, postpartum hemorrhage; DIC, disseminated intravascular coagulation; ICU, intensive care unit; SMM severe maternal morbidity

AOR adjusted for maternal age ( <25yrs, 25-35yrs, ≥35yrs), maternal education (high school graduation or higher vs less than high school graduation), marital status (single, widowed, or separated vs married or common law), race/ethnicity (Hispanic, African American, Native American, and other vs non-Hispanic white), parity (nulliparous, parity ≥4 vs parity 1-3), assisted conception (no vs yes), smoking during pregnancy (no vs yes), type of health insurance (Medicaid, private vs other), year of birth, and fetal sex (female vs male).
