# Supplementary material for: Association between gestational weight gain and severe adverse birth outcomes in Washington State, US: A population-based retrospective cohort study, 2004–2013
Source: PLoS Med. 2019 Dec 30;16(12):e1003009. doi: 10.1371/journal.pmed.1003009 (PMC6936783; doi:10.1371/journal.pmed.1003009)
Supplement: S7 Table — (DOCX) [file pmed.1003009.s009.docx]

**S7 Table**. Adjusted Odds Ratios (AOR) for severe adverse maternal and perinatal outcomes by gestational weight gain and pre-pregnancy Body Mass Index, singleton births **at term gestation (≥37 weeks)**, Washington State, 2004-2013 (AOR relative to optimal weight gain in each pre-pregnancy Body Mass Index category).

| **Pre-pregnancy BMI** | **Pre-pregnancy**  **Underweight** | | **Pre-pregnancy**  **Normal weight** | | **Pre-pregnancy**  **Overweight** | | **Pre-pregnancy**  **Obese** | |
| --- | --- | --- | --- | --- | --- | --- | --- | --- |
| **Outcomes** | **L-GWG**  AOR  (95% CI)  *P*-value^±^ | **E-GWG**  AOR  (95% CI)  *P*-value^±^ | **L-GWG**  AOR  (95% CI)  *P*-value^±^ | **E-GWG**  AOR  (95% CI)  *P*-value^±^ | **L-GWG**  AOR  (95% CI)  *P*-value^±^ | **E-GWG**  AOR  (95% CI)  *P*-value^±^ | **L-GWG**  AOR  (95% CI)  *P*-value^±^ | **E-GWG**  AOR  (95% CI)  *P*-value^±^ |
| **Maternal death/SMM** | 0.84  (0.62-1.13)  *1* | 1.24  (0.94-1.64)  *1* | **1.11**  **(1.02-1.21)**  *0.01* | **1.14**  **(1.06-1.23)**  *<0.001* | 1.06  (0.92-1.21)  *0.366* | 1.01  (0.92-1.12)  *0.755* | 1.03  (0.91-1.17)  *0.668* | **1.12**  **(1.01-1.25)**  *0.034* |
| **Perinatal death/Severe neonatal morbidity** | 0.10  (0.67-1.58)  *0.901* | 1.12  (0.74-1.69)  *0.621* | 1.03  (0.92-1.15)  *0.617* | 1.08  (0.99-1.19)  *0.092* | 1.11  (0.93-1.34)  *0.176* | 0.99  (0.87-1.13)  *0.872* | **1.25**  **(1.07-1.46)**  *0.003* | **1.19**  **(1.04-1.36)**  *0.009* |

Abbreviation: O-GWG, optimal gestational weight gain; L-GWG low gestational weight gain; E-GWG, excess gestational weight gain

AOR Adjusted for maternal age ( <25yrs, 25-35yrs, ≥35yrs), maternal education (high school graduation or higher vs less than high school graduation), marital status (single, widowed, or separated vs married or common law), race/ethnicity (Hispanic, African American, Native American, and other vs non-Hispanic white), parity (nulliparous, parity ≥4 vs parity 1-3), assisted conception (no vs yes), smoking during pregnancy (no vs yes), type of health insurance (Medicaid, private vs other), year of birth, and fetal sex (female vs male).

^±^2-sided p-values were calculated using multivariable logistic regressions Wald Chi-square test.
