# Supplementary material for: Association between gestational weight gain and severe adverse birth outcomes in Washington State, US: A population-based retrospective cohort study, 2004–2013
Source: PLoS Med. 2019 Dec 30;16(12):e1003009. doi: 10.1371/journal.pmed.1003009 (PMC6936783; doi:10.1371/journal.pmed.1003009)
Supplement: S1 Text — (DOCX) [file pmed.1003009.s010.docx]

# S1 Text. Study Analyses Plan (REB proposal)

Sarka Lisonkova, MD, PhD June 29, 2015

# Pregnancy outcomes and severe maternal morbidity – temporal trends

**Background**

Maternal health is an international priority as one of the Millennium Development Goals set by United Nations is to reduces maternal mortality ratio by three quarters by 2015.1 In the USA, the pregnancy-related mortality ratio (11.8 deaths per 100,000 live births) has not improved in past 2 decades.2,3 Besides monitoring of temporal trends in maternal mortality ratio, it is important to examine trends in severe maternal morbidity, as maternal death typically represent only a small portion of adverse maternal birth outcomes. However, the population rates of severe maternal morbidity are difficult to estimate, because information on these adverse events is not routinely collected and complete population data are required to avoid selection bias typically present in hospital-based studies. As a result, relatively little is known about the temporal trend in severe maternal morbidity and its determinants. Although some medical conditions and their associated morbidity and death are not avoidable, the progression of disease may be prevented through changes in medical care. Some studies suggest that 40-50% of maternal deaths and 30-40% of ‘near-miss’ or severe morbidities are preventable.4-9 In addition, severe maternal morbidity profoundly affects the birth outcomes and fetal and neonatal health. Temporal changes in the association between severe maternal morbidity and perinatal mortality/morbidity has not been examined to date.

This study proposes to use large linked population databases to examine the temporal trend in severe maternal morbidity, and its determinants including maternal age, marital status, body- mass-index and chronic diseases.

# Objective.

1. To examine trend in severe maternal morbidity between 1987 and 2011 (or most recent year) and its association with perinatal mortality and morbidity.
2. To examine determinants of severe maternal morbidity including maternal age, marital status, body-mass-index and chronic diseases.

# Materials and Methods

Ethics approval for this study will be obtained from the institutional research ethics board.

*Study population*

1. All women of reproductive age (15-55 years) who were hospitalized in Washington state for pregnancy related condition from 1987 to 2013 (or the most recent year with available data).
2. All women who delivered in Washington state between 1987 and 2013 (or the most recent year with available data) and their infants.

*Methods and Data*

1. We will use the Washington state’s Comprehensive Hospital Abstract Reporting System database (CHARS) that includes information on all hospitalizations from 1987-most recent. CHARS data contain information on patient’s age, residence, type of admission (urgent vs. elective), and ICD-9 codes for up to 25 diagnoses and procedures related to the hospitalization. Women with pregnancy complication related to early pregnancy (before 20 weeks gestation) will be identified from CHARS by ICD-9 codes (Appendix 1).
2. Women with pregnancy complication and morbidity after 20 weeks gestation will be identified from Washington State Linked Birth-CHARS file (1987-most recent). Definition of pregnancy complications and maternal morbidity arising from pregnancy beyond 20 weeks gestation are included in Appendix 2, perinatal outcomes are listed in Appendix 3. Preterm birth will be identified as a birth at < 37 weeks gestation, we will use the clinical estimate of gestation provided in the data source as this is more accurate than gestational age estimated by the last menstrual period.

BIRTH-CHARS linked files include also information on maternal demographic characteristics, pre-existing maternal morbidity, and obstetrical history which will be used to evaluate potential confounders. These include maternal age, parity (number of previous live births, none vs. ≥1), marital status (single/widowed/separated vs. married/common law), education (less than high school vs. high school education or greater), BMI, race (non-Hispanic White vs. Hispanic,

African-American, Native-American and other), smoking during pregnancy (yes/no), infertility treatment (yes/no), diabetes mellitus (yes/no), chronic hypertension prior to pregnancy (yes/no), infant’s sex (male/female), and congenital anomalies (yes/no).

*Statistical analysis*

Cochran-Armitage test for trend will be used to assess temporal trends. Logistic regression will be used to adjust for temporal trends in maternal risk factors. Temporal changes in population attributable fraction for major risk factors for maternal morbidity will be also examined.

All analyses will be carried out using SAS-PC statistical software version 9.3 (SAS Inc., Cary NC).

# Rationale

Large population data are needed to identify temporal trends and determinants of relatively rare events, such as severe maternal morbidity. This information will provide population perspective on an important public health issue and help to guide preventive efforts.

# References:

1. <http://www.un.org/millenniumgoals/maternal.shtml>Accessed June 2015
2. Abou Zahr C. Maternal mortality in 2000: Estimates developed by WHO, UNICEF and UNFPA. Geneva: Department of Reproductive Health and Research, World Health Organization; 2004.
3. Chang J, Elam-Evans LD, Berg CJ, et al. Pregnancy-related mortality surveillance: United States, 1991-1999. MMWR Surveill Summ 2003;52:1-8.
4. 4. Geller SE, Rosenberg D, Cox SM, et al. The continuum of maternal morbidity and mortality: Factors associated with severity. Am J Obstet Gynecol 2004;191:939-44.
5. Berg CJ, Harper MA, Atkinson SM, et al. Preventability of pregnancy-related deaths: Results of a state-wide review. Obstet Gynecol 2005; 106:1228-34.
6. Nannini A, Weiss J, Goldstein R, Fogerty S. Pregnancy-associated mortality at the end of the twentieth century: Massachusetts, 1990-1999. J Am Med Womens Assoc 2002;57:140- 3.
7. Panting-Kemp A, Geller S, Nguyen T, Simonson L, Nuwayhid B, Castro L. Maternal deaths in an urban perinatal network, 1992-1998. Am J Obstet Gynecol 2000;183:1207-12.
8. Berg CJ, Atrash HK, Koonin LM, Tucker M. Pregnancy-related mortality in the United States, 1987-1990. Obstet Gynecol 1996;88:161-7.
9. Hoyert DL, Danel I, Tully P. Maternal mortality, United States and Canada, 1982-1997. Birth 2000;27:4-11.

# Appendix 1:

630- 633 ectopic and molar pregnancy 634-639 pregnancy with abortive outcome 640 Hemorrhage in early pregnancy

**Appendix 2:** Diagnostic codes for pregnancy complications and severe maternal morbidity at 20 weeks gestation or more:

| **Maternal morbidity:** | **ICD-9** | **Diagnosis** |
| --- | --- | --- |
| Antepartum hemorrhage | 661.0 | placenta previa |
|  | 661.1 | placenta previa, includes accidental antepartum hemorrhage |
|  | 641.2 | placental abruption |
|  | 641.1 | hemorrhage from placenta |
|  | 641.3 | antepartum hemorrhage associated with coagulation defects |
|  | 641.8 | other |
|  | 641.9 | unspecified |
| Preeclampsia | 642.4 | mild or unspecified preeclampsia |
|  | 642.5 | severe preeclampsia |
|  | 642.6 | eclampsia |
|  | 642.7 | preeclampsia/eclampsia superimposed on pre-existing hypertension |
| Respiratory morbidity | 673 | obstetric pulmonary embolism |
|  | 673.1 | amniotic fluid embolism |
|  | 518.0 | pumonary collapse |
|  | 518.4 | acute oedema |
|  | 518.5 | shock - lung |
|  | 518.8 | adult RDS |
|  | 415.0 | acute cor pulmonale |
|  | 415.1 | pulmonary embolism (septic, iatrogenic, infection, other) |
| Thromboembolism | 671.5 | cerebral venous thrombosis |
|  | 671.3 | deep phlebothrombosis - antepartum |
|  | 671.4 | deep phlebothrombosis - postpartum |
|  | 444.0 | arterial embolism and thrombosis of abdominal aorta |
|  | 444.1 | embolism or thrombosis of thoracic aorta |
|  | 673.2 | pulmonary thrombosis - clot |
| Cardiovascular morbidity |  |  |
|  | 674.5 | peripartum cardiomyopathy |
|  | 671.0 | cerebrovascular disorders |
|  | 428 | heart failure |
|  | 427.3 | atrial fibrilation/flutter |
|  | 427.4 | ventricular |
|  | 410 | acute myocardial infarction |
|  | 427.5 | cardiac arrest |
|  | 423.0 | hemopericardium |
|  | 423.2 | constrictive percarditis |
|  | 423.3 | cardiac tamponade |
|  | 4010 | malignant essential hypertension |
|  | 4020 | malignant essential hypertension with kidney involvement |

|  | 4030 | malignant hypertension |
| --- | --- | --- |
|  | 4040 | malignant hypertension with kidney involvement |
|  | 444 | arterial embolism (abdominal , thoracic aorta) |
|  | 441 | aortic aneurism and dissection |
|  | 417.1 | aneurism of pulmonary artery |
|  | 4233 | tamponade |
|  | 4171 | aneurism of pulmonary artery |
| Central nervous system  morbidity | 430 | subarchnoid hemorrhage |
|  | 431 | intracerebral hemorrhage |
|  | 432.1 | subdural hemorrhage |
|  | 432.9 | unspecified intracranial hemorrhage |
|  | 433 | occlusion or stenosis of pre-cerebral arteries |
|  | 434 | occlusion or stenosis of cerebral arteries |
|  | 436 | cerebral seizure, apoplexy |
|  | 348.3 | encephalopathy |
|  | 344 | other paralytic syndromes |
|  | 342 | hemiplegia, hemiparesis |
|  | 674.0 | cerebrovascular disorders of puerperium |
| Acute renal failure | 584 | acute and subacute renal failure |
|  | 586 | renal failure, unspecified |
|  | 669.3 | other renal failure |
| Acute liver failure | 570 | acute and subacute liver failure |
| Infection/sepsis |  |  |
|  | 038 | septicaemia |
|  | 659.3 | septicaemia during labor |
|  | 639.0 | genital tract and pelvic infection (abortive outcome) |
|  | 785.5 | septic shock |
|  | 995.9 | systemic inflammatory response syndrome (SIRS) |
|  | 670 | major puerperal infection |
| Postpartum hemorrhage | 666 | postpartum hemorrhage |
|  | 758 | procedure code obstetric tamponade of uterus and vagina |
| Complications of anesthesia |  |  |
|  | 668.0 | pulmonary complications |
|  | 668.1 | cardiac complications |
|  | 668.2 | CNS complications |
|  | 669.4 | other complications of surgery |
|  | 995.0 | other anaphylactic shock |
|  | 995.4 | shock due to anesthesia |
|  | 997.01 | CNS complications (anoxic brain damage, cerebral hypoxia) |
|  | 997.02 | iatrogenic cerebrovascular infarction (postoperative stroke) |
|  | 997.1 | cardiac complications (during procedure) |
|  | 998.0 | postoperative shock |

|  | 665.1 | rupture of uterus during labor |
| --- | --- | --- |
| Other (DIC, complications of  obstetric procedures, etc.) | | |
|  | 679.0 | maternal complications from in-utero procedures |
|  | 286.6 | disseminated intravascular coagulation |
|  | 669.1 | obstetric shock |
| Hysterectomy | procedure codes 68.3-68.4, 68.6 | |
| Transfusion (any blood  products) | procedure codes 99.0 | |
| Obstetric trauma | 665.0 | rupture of uterus before onset of labor |
|  | 665.1 | rupture of uterus during labor |
|  | 665.2 | inversion of uterus |
|  | 665.3 | laceration of cervix |
|  | 665.4 | high vaginal laceration |
|  | 665.5 | other injury to pelvic organs |
|  | 665.6 | damage to pelvic joints and ligaments |
|  | 664.2 | perineal tear 3rd |
|  | 664.3 | perineal tear 4th |
|  | 664.6 | anal sphincter tear |

**Appendix 3**: List of neonatal adverse outcomes:

Fetal death: in-utero or intra-partum death of a fetus delivered at 20 weeks gestation or later. Neonatal death: death of an infant within 28 days after birth.

Perinatal death: fetal or neonatal death. Bronchopulmonary dysplasia (BPD, code 770.7).

Intraventricular haemorrhage (IVH) grade III and IV (codes 772.13 and 772.14). Periventricular leukomalacia (PVL, code 779.7).

Retinopathy of prematurity (ROP, code 362.2). Necrotizing enterocolitis (NEC, code 777.5).

Neonatal sepsis (code 771.81).

Severe trauma (code 767.0, 767.4, 767.11).

Small-for-gestational-age (SGA): infants weighing less than the 10th percentile of the sex- and gestational age- specific birth weight reference for the United States.12

Large-for-gestational-age: infants weighing over the 90th percentile.

Other neonatal outcomes will be identified from birth records, namely, neonatal seizures, Apgar score at 5 minutes ≤3, and NICU admission.

Severe neonatal morbidity will include any of the following: neonatal seizures, BPD, IVH grade III or IV/PVL, ROP, NEC, severe trauma and neonatal sepsis.
