# Supplementary material for: Association between gestational weight gain and severe adverse birth outcomes in Washington State, US: A population-based retrospective cohort study, 2004–2013
Source: PLoS Med. 2019 Dec 30;16(12):e1003009. doi: 10.1371/journal.pmed.1003009 (PMC6936783; doi:10.1371/journal.pmed.1003009)
Supplement: Attachment — Submitted filename: Response to editors and reviewers_final.docx [file pmed.1003009.s011.docx]

**Response to the editors and reviewers:**

-----------------------------------------------------------

We thank editors and reviewers for their thoughtful comments to improve our manuscript (PMEDICINE-D-19-02499R1 - [EMID:8961d287660f9a89]). We have tried to address them in the manuscripts and responded to comments below. We will also be happy to address any further comments if required.

**Requests from the editors:**

1. Title – please add a study design in title, and remove rhetorical question

**Response:** The title was changed to: “Association between gestational weight gain and severe adverse birth outcomes: A population-based retrospective cohort study”

2. Please comment on the limitation of the data and how old it is.

**Response:** We added the limitation to the Abstract as follows:

Line 59-60: “Study limitations include the ascertainment of pre-pregnancy BMI using self-report, and lack of data availability for the most recent years.”

3. Please provide a summary of demographic details and limitations in abstract (limitations as the final sentence of the Methods and Findings section.)

**Response:** We included summary of demographic details on Lines 45-48 as follows:

“Women who had low weight gain were more likely to be African American and have Medicaid health insurance, while women with excess weight gain were more likely to be Non-Hispanic White, and younger, as compared with women with optimal weight gain in each pre-pregnancy BMI category.”

4. Please provide p values where 95%Cis are given.

**Response:** We have included P values for all 95% CIs in the abstract (Lines 52-55) and throughout the main manuscript text and tables.

5. Data – please be explicit about why access to data is prohibited (IRB or ethical restrictions, for example).Also we need the email or URL for (eligible) people to access data. Noting this cannot be an author.

**Response:** Data are available upon request from the Department of Health, Washington State, USA. <https://www.doh.wa.gov/DataandStatisticalReports/HealthcareinWashington/HospitalandPatientData/HospitalDischargeDataCHARS>

We have also included this information on the PLOS MED website submission details.

6. At this stage, we ask that you include a short, non-technical Author Summary of your research to make findings accessible to a wide audience that includes both scientists and non-scientists. The Author Summary should immediately follow the Abstract in your revised manuscript. This text is subject to editorial change and should be distinct from the scientific abstract. Please see our author guidelines for more information: <https://journals.plos.org/plosmedicine/s/revising-your-manuscript#loc-author-summary>

**Response:** We have included the Author Summary in the revised manuscript, following the abstract; Lines 69 to 101.

7. Ethics – please say why it was exempt.

**Response:** According to the Washington State Institutional Review Board (Exemption Determination Request Form, SECTION 3: Is the Research Exempt from WSIRB Review?), a research study can be exempt from the Washington State Institutional Review Board review if “the research does not involve obtaining data about subjects through interaction or intervention with individuals, and does not involve obtaining identifiable private information about subjects from confidential records.”

Our study was granted this exemption (DOH Exempt Request E-050515-H; contact person: Phyllis.Reed@DOH.WA.GOV).

We have also included this information on the PLOS MED website submission details.

8. Did your study have a prospective protocol or analysis plan? Please state this (either way) early in the Methods section.

a) If a prospective analysis plan (from your funding proposal, IRB or other ethics committee submission, study protocol, or other planning document written before analyzing the data) was used in designing the study, please include the relevant prospectively written document with your revised manuscript as a Supporting Information file to be published alongside your study, and cite it in the Methods section. A legend for this file should be included at the end of your manuscript.

b) If no such document exists, please make sure that the Methods section transparently describes when analyses were planned, and when/why any data-driven changes to analyses took place.

c) In either case, changes in the analysis—including those made in response to peer review comments—should be identified as such in the Methods section of the paper, with rationale.

**Response:** A prospective analysis plan was created prior to data request to the Washington State Department of Health. The project (‘Pregnancy outcomes and severe maternal morbidity’) description was broad and included several objectives, one of which was to examine risk factors for adverse maternal and perinatal outcomes, such as BMI (including weight gain during pregnancy). A specific plan for the data analysis was developed before the analysis. There were no data-driven changes to our analyses. This information was added to the manuscript:

Line 142-143: "The study methodology and analysis plan were developed prior to data manipulation and the actual analysis."

We have also included our document as a separate file in the submission (please see REB proposal under “other” in the submission file).

9. Please ensure that the study is reported according to the [STROBE] guideline, and include the completed [STROBE or other] checklist as Supporting Information. When completing the checklist, please use section and paragraph numbers, rather than page numbers. Please add the following statement, or similar, to the Methods: "This study is reported as per the Strengthening the Reporting of Observational Studies in Epidemiology (STROBE) guideline (S1 Checklist)."

**Response:** We completed the STROBE checklist, included as an Appendix S1, and we added the following statement to the Methods sections:

Line 141: “This study is reported according to the Strengthening the Reporting of Observational Studies in Epidemiology (STROBE) guideline (see Checklist in S1).”

10. References should be in square brackets instead of round throughout text.

**Response:** This has been changed consistently throughout the manuscript.

11. Line 397 – I wonder if “We did not aim to examine causal associations” should be removed. As this is an observational study, causality couldn’t be assessed anyway. Otherwise rephrase to outline that this is not possible in this study.

**Response:** This line has been removed from the text.

12. Please use the "Vancouver" style for reference formatting, and see our website for other reference guidelines <https://journals.plos.org/plosmedicine/s/submission-guidelines#loc-references>

**Response:** The reference style has been changed as requested.

Comments from the reviewers:

**Reviewer #1:**

14.	This is a well-conducted study on the association between gestational weight gain and severe adverse birth outcomes, analysed in different pre-pregnancy BMI categories. The study design, datasets, statistical methods and analyses, and presentation (tables and figures) and interpretations of results are mostly adequate and of a good standard. Only a couple of minor points needing attention:

Table 2 and 3 are massive tables with a lot of details which are not as important as other tables in terms of primary analyses. To be concise, suggest to remove both or one of the tables to the supplementary information.

**Response:** We have moved table 3 to the supplementary section.

15.	Table 1 is a huge descriptive table but without any statistical test. I know it's already huge, but it would be helpful to add a column of p-values in each of the pre-pregnancy BMI categories to test the statistical significance of the baseline characteristics across the levels of weight gain, which will make it easy for the readers to read and follow the main message.

**Response:** We have thought about a reasonable solution to accommodate this suggestion. In general, there is a trend towards not including p-values in Table 1, as they depend largely on the size of the study population and Table 1 is merely descriptive of the population characteristics. However, we have included the additional p-values from table 1 at the end of this document (see below). If there is a consensus on including this information as an Appendix table, we can add that.

--------------------------------------------------------------------------------------------------------------------------------

**Reviewer #2:**

16.	This large observational study shows adverse health impacts of inadequate or excess gestational weight gain, with a focus on severe morbidity and mortality. Linked data sets and sheer size of the analysis are strengths of this work. However, the limitations need also to be addressed.

The findings are largely consistent with prior literature. The clear gaps proposed in the introduction are not strongly justified. The studies referenced in the introduction have previously addressed many of the key gaps in this field and the contribution of prior literature and the very specific nuances aims and hypotheses should be more clearly outlined. It is not clear what the specific initial hypotheses were and why?

**Response:** We agree that there have been several publications focusing on gestational weight gain and birth outcomes, however, our study includes several strengths as pointed out by the reviewer #3, for instance: “the large number of cases could easily serve as a reference manuscript. The inclusion of both pre-term and term pregnancies, along with detailed information about class I, II, and III obesity, are important components of this manuscript. As noted by the authors in the introduction, there was a lack of specific recommendations relating to gestational weight gain in the IOM report in class I, II and III obese women because of the lack of data in 2008. The information in this manuscript helps to improve that knowledge gap.”

First, the majority of previous studies (references 8-9 and 14-15) did not include preterm deliveries, and therefore underestimate the overall association between suboptimal weigh gain and adverse pregnancy outcomes. We made it clearer in the manuscript.

Line 114: “And while the suboptimal weight gain is associated with preterm delivery,(5,10-13) majority of studies on gestational weight gain included only term pregnancies,( 8-9; 14-15) which largely underestimates the overall association between suboptimal weight gain and adverse pregnancy outcomes .

Second, while many prior studies examined adverse birth outcomes that are less severe, e.g., SGA, LGA, caesarean delivery, only few studies focussed on severe morbidity with potentially serious sequelae. Even the study by Voerman et al (JAMA 2019) which was published after we submitted the manuscript, included less severe adverse maternal morbidity such as preeclampsia, gestational hypertension and gestational diabetes, which are typically not life-threatening. None of these studies included both maternal and fetal/infant severe morbidity. This is important because women and their caregivers need to know the incidence of *any* serious complication. We added the following text:

Line 124: “None of the prior studies included all serious adverse birth outcomes, including death and severe maternal and fetal/infant morbidity.”

Third: One study examined the association between gestational weight gain and severe maternal morbidity (Platner *et al,* reference 16). This study did not stratify by BMI category, except for reporting the composite maternal outcome (severe maternal morbidity, SMM). This study included only term pregnancies, resulting in the underestimation the overall association between suboptimal weight gain and SMM.

Fourth: The association between gestational weight gain and adverse birth outcomes among women with class I, II and III obesity prior to pregnancy was investigated by several studies, but was not addressed in a comprehensive manner, including preterm births and all severe maternal and fetal/infant health outcomes.

17. It is not clear why ethics review was waivered and this should be further clarified, especially given the use of linked data bases.

**Response:** We have now addressed this; Please, see the response to comment #7 regarding ethics.

18. A clear rationale for the composite outcomes should be provided.

**Response:** the composite outcome of severe maternal morbidity included severe maternal conditions that were identified by the Canadian Perinatal Surveillance system, and included conditions that are potentially life-threatening, may have long-term sequelae, or associated with organ damage and prolonged hospitalization (more details included in reference 24). In addition we included conditions recognized as severe maternal morbidity by the Centre for Disease Control and Prevention (CDC), USA, if they were not already included in the list of SMM developed by the Canadian Perinatal Surveillance system. The Methods section:

Line 204: “SMM included maternal conditions that had high case-fatality rate or those leading to a vital organ damage or serious long-term sequelae. A list of such conditions was developed by the Canadian Perinatal Surveillance system.(24) In addition, we included conditions recognized as severe maternal morbidity by the Centre for Disease Control and Prevention (CDC), USA,(25-27) for instance, life-saving procedures such as mechanical ventilation, conversion of cardiac rhythm, etc. (see full list and definitions in Appendix Table S2).”

With respect to severe neonatal morbidity, the composite outcome included conditions with high mortality and those with high risk of neurodevelopmental disability. A composite outcome including bronchopulmonary dysplasia, respiratory distress syndrome, retinopathy of prematurity, intraventricular hemorrhage grade 3 or more, periventricular leukomalacia, neonatal sepsis, necrotizing enterocolitis, severe birth trauma, and neonatal seizures has been used extensively in the past literature (see below).

1) Edstedt Bonamy AK, Zeitlin J, Piedvache A on behalf of Epice Research Group, et al. Wide variation in severe neonatal morbidity among very preterm infants in European regions. Archives of Disease in Childhood - Fetal and Neonatal Edition 2019;104:F36-F45.
2) Lisonkova S, Hutcheon JA, Joseph KS. Temporal trends in neonatal outcomes following iatrogenic preterm delivery. BMC Pregnancy and Childbirth 2011;11:39.
3) Richter LL, Ting J, Muraca GM, et al. Temporal trends in neonatal mortality and morbidity following spontaneous and clinician-initiated preterm birth in Washington State, USA: a population based study. BMJ Open 2019;9:e023004. doi:10.1136/bmjopen-2018-023004
4) Lisonkova S, Potts J, Muraca GM, Razaz N, Sabr Y, Chan WS, Kramer MS. Maternal age and severe maternal morbidity: population-based study. PloS Medicine 2017;14(5): e1002307. https://doi.org/10.1371/journal.pmed.1002307.
5) Richter L. et al. Temporal Trends in Preterm Birth, Neonatal Mortality, and Neonatal Morbidity Following Spontaneous and Clinician-Initiated Delivery in Canada, 2009–2016 Journal of Obstetrics and Gynaecology Canada
6) Lisonkova S, Richter L, Ting J, Muraca G, Wen Q, Mehrabadi A, Mitchell-Foster S, Oviedo-Joekes E, Lyons J. Maternal and neonatal mortality and severe morbidity associated with neonatal abstinence syndrome. Pediatrics 2019; 144(2):e20183664. https://doi.org/10.1542/peds.2018-3664
19. The observation on line 221 that those with GDM were more likely to have low GWG is expected as treatment of GDM reduces maternal weight gain. This presents an interesting subgroup to study relationships between GWG and outcomes and could perhaps be explored as a subgroup here.

**Response:** We agree that this is an interesting subgroup, as pointed out also by the reviewer #3. We highlighted this finding in the Discussion, however, a full subgroup analysis is beyond the scope of this paper. As this was not our primary objective, this analysis would be completely data-driven.

20. The data presented here reflects the nuances of the specific ethnicities in the US and the unique health system in the US, both of which are limitations for generalisability. This is especially regarding the health system which requires around 20% GDP and has significant access and disparity issues, limiting generalisability and this should be acknowledged. Likewise the ethnic variation shown to vary in Asian subgroups specifically (eg Goldstein et al BMC Medicine), limits the broader generalisability for example in the majority of the international population who are of Asian ethnicity.

**Response:** We agree that any population-based study will to some extent reflect the characteristics of the population being studied. We adjusted for race/ethnicity and for the type of health insurance to limit the confounding effect of these characteristics. We agree, that the proportion of women with suboptimal weight gain vary across populations, however, the effect of suboptimal weight gain has been shown to be quite robust by Goldstein et al BMC Medicine, as this study concluded “GWG outside guidelines is associated with adverse outcomes across all regions.” The outcomes that were compared across regions were: SGA, LGA and preterm birth, secondary outcomes were macrosomia, caesarean section and gestational diabetes. We added the following text to the Discussion section about limitations:

Line 471: “Last, our finding that more than a half of women did not achieve optimal weight gain during pregnancy may be influenced by the ethnic/racial composition and other characteristics of pregnant women in Washington State. And while similar proportions have been observed in the USA and Europe, in Asian countries, for instance, the proportion of women with excess weight gain is much lower when standard BMI categories are applied. [31]”

We added the reference: 31 (Goldstein et al BMC Medicine)

21. Table 4 is difficult to interpret, as there seems to be a separate column of data superimposed on the last column.

**Response:** This unfortunate shift likely occurred during the file transfer. We apologise for the lack of clarity; this has been corrected.

22. The focus on the inconsistent findings of rare outcomes across multiple subgroups as noted in the first paragraph on the discussion, are difficult to interpret and appear inconsistent. For example findings in normal and obese but not overweight women. These subgroup results appear inconsistent and not driven by plausible hypotheses, an area that needs to be more thoroughly explored.

**Response:** We modified the first paragraph in the Discussion to avoid the excess description of subgroup findings as follows:

Line 393: “Low weight gain was consistently associated with increased risk of severe adverse birth outcomes, and in particular with maternal death and perinatal death. On the other hand, excess weight gain was associated with adverse maternal and perinatal outcomes only in women who had pre-pregnancy BMI in the normal range and below. Women who were overweight prior to pregnancy and gained excess weight during pregnancy did not have elevated risks of serious adverse birth outcomes as compared with overweight women with optimal weight gain. Pre-pregnancy obesity and excess weight gain was associated with elevated risk of SMM.”

23. Broader existing literature, evidence synthesis and meta-analyses need to be considered in the discussion. For example the discussion in lines 373-377 should be revised to extend to evidence that does not support these findings including meta analyses and the potential reasons why. Specifically the differences from JAMA. 2019;321(17):1702-1715. doi:10.1001/jama.2019.3820 and JAMA. 2017;317(21):2207-2225. doi:10.1001/jama.2017.3635 as large scale intergrated evidence synthesis studies and the findings here should be discussed in this context.

**Response:** We included findings of two meta-analyses in the Discussion section, however, the focus of these two meta-analyses was on less severe birth outcomes, namely, SGA, LGA, macrosomia, preterm birth, preeclampsia, gestational diabetes, gestational hypertension and caesarean delivery. Cesarean delivery, in particular, is not considered as an ‘adverse outcome’ in our study, rather, cesarean delivery can *prevent* some of the severe adverse maternal and perinatal outcomes such as stillbirth, eclampsia, uterine rupture, etc. In that view, higher rates of caesarean delivery can be associated with lower rates of SMM in women with high pre-pregnancy BMI and excess weight gain. For these reasons, it is difficult to draw direct comparisons between these meta-analyses and our study. However, we discuss these important papers in the light of our results with respect to SGA and LGA.

We added the following text to the Discussion:

Line 410: “Two large meta-analyses focussing on less severe adverse birth outcomes showed elevated rates of LGA and macrosomia in women with excess weight gain in all pre-pregnancy BMI categories, and elevated rates of SGA in women with low weight gain,[2,31] which is consistent with our results.”

--------------------------------------------------------------------------------------------------------------------------------

**Reviewer #3:**

24. The purpose of this study was to investigate the association between suboptimal and excessive weight gain and the effects on adverse birth outcomes in women with a range of pre-pregnancy BMI. This was a population-based study of a singleton hospital births in the state of Washington between 2004 and 2013. The primary composite outcome included: 1) maternal death or severe maternal morbidity, and 2) perinatal death and/or severe neonatal morbidity. Overall 722,839 women with information on pre-pregnancy BMI were included in the analysis. The distribution of BMI weight classes, according to WHO criteria, was consistent with what has been previously reported. Low gestational weight gain was associated with higher rates of maternal death and severe maternal morbidity in women with normal pre-pregnancy BMI and overweight women. Similarly, excessive gestational weight gain was associated with an increased rate of death and severe maternal morbidity among women with normal pre-pregnancy BMI and obese women. Low gestational weight gain was associated with perinatal death and severe neonatal morbidity regardless of pre-pregnancy BMI, including obese classes 1, 2, and 3. The authors conclude that most women do not achieve optimal weight during pregnancy. Both low and excessive gestational weight gain were associated with severe adverse birth outcomes regardless of pre-pregnancy BMI except for overweight women with excessive gestational weight gain.

Specific Comments:

This is a well-written manuscript with detailed tables, which because of the large number of cases could easily serve as a reference manuscript. The inclusion of both pre-term and term pregnancies, along with detailed information about class I, II, and III obesity, are important components of this manuscript.

As noted by the authors in the introduction, there was a lack of specific recommendations relating to gestational weight gain in the IOM report in class I, II and III obese women because of the lack of data in 2008. The information in this manuscript helps to improve that knowledge gap.

Gestational age at delivery was based on ultrasound dating. One assumes that the ultrasound dating in the vast majority of subjects was done in early pregnancy. It would be helpful if the authors would provide information on gestational age at the time of the ultrasound.

**Response:** We did not have information on timing of ultrasound, however, the first ultrasound is usually done during the first trimester.

25.	Pregnancy weight gain was defined as a woman's weight measured at childbirth subtracted from her pre-pregnancy self-reported weight. Many investigators use weight at the last ante-natal visit as the end point of weight gain during pregnancy. In the state of Washington, was maternal weight actually measured in the labor and delivery unit?

**Response:** Information on weight at birth was obtained from the Washington State Birth filling form (a revision of this form was undertaken in 2003). This information is abstracted from medical charts and retained in the Birth form (“Mother’s weight at delivery”). This would correspond to maternal weight at the hospital admission for delivery.

26.	To account for gestational age at delivery among women who delivered pre-term, weight pre-term was calculated using the IOM criteria. These criteria provide a range of weight gain per week. Which part of the range was used in the analysis and was it used consistently among the various BMI groups?

**Response:** The IOM provides recommendation for weight gain per week and its range for each pre-pregnancy BMI group, so that weight gain can be calculated per week during the preterm gestation. This is under an assumption of weight gain between 1.1 and 4.4 lbs during the first trimester.

For example, if a woman had a pre-pregnancy underweight BMI and delivered at 26 weeks gestation (13 weeks after first trimester), optimal weight gain would be between [1.1 + (13*1)] AND [4.4 + (13*1.3)]; i.e. 14.1-21.3 lbs. Any weight gain below or above would be considered as low and excess weight gain respectively.

This was done consistently for all preterm deliveries for pre-pregnancy BMI groups using the recommended range for each group. We added the following to the Methods:

Line 195: “These recommendations pertain to the second trimester, assuming a weight gain range of 1.1-4.4 lbs in the first trimester, all calculations were performed consistently for each BMI category.(1)”

27.	Very detailed information is provided in both the tables in the manuscript and in the supplementary tables. Is all of this this information available in the BERD and CHARS data bases or were additional means used to attain the granularity of detail presented in these tables?

**Response:** All information was obtained from the BERD and CHARS datasets. We attached the Washington State Birth filling form (2003 revision) as a supplementary material (S9) to provide detailed information on the majority of data (obtained from BERD).

28.	The percentage of women in each pre-pregnancy BMI weight category is pretty much consistent as what has been reported in the literature, including data from the CDC.

**Response:** This suggests a relatively good ascertainment of pre-pregnancy BMI in our study.

29.	On the bottom of page 11, women with low weight gain had higher rates of spontaneous vaginal delivery and lower rates of prolonged labor. The rates of gestational diabetes were higher in these women, especially for those who were underweight and those with a normal BMI prior to pregnancy. These data are very interesting since many previous studies have related the increased risk of gestational diabetes on excessive gestational weight gain and not low gestational weight gain.

**Response:** We agree that this was an interesting finding, and we discussed that in the Discussion. As mentioned by the reviewer #2 (point #19), it is possible that the treatment of gestational diabetes may have, in part, contributed to low gestational weight gain.

30.	The data presented in table 3 at the bottom of page 18 are important as noting that low gestational weight gain had a significantly higher rate of maternal death as compared with women with optimal weight gain (7.97 vs. 2.63 per 100,000 p=0.027). Among normal weight and overweight women, those with low gestational weight gain had higher adjusted odds of maternal death and severe morbidity compared with optimal gestational weight gain. These data are important to note given that many individuals recommended gestational weight gain less than IOM guidelines to improve some perinatal outcomes.

**Response:** We agree this is an important finding. We mentioned this in the Results, and we added this finding in the Abstract:

Line 48: “Compared with women who had optimal weight gain, those with low gestational weight gain had higher rates of maternal death, 7.97 vs. 2.63 per 100,000 (p=0.027).”

“Low weight gain was consistently associated with increased risk of severe adverse birth outcomes, and in particular with maternal death and perinatal death.”

Author Summary:

Line 85: “Low gestational weight gain was associated with an increase in adverse perinatal outcomes, particularly with maternal death and stillbirth; and with an increase in severe maternal morbidity in women with normal pre-pregnancy BMI and overweight women.”

And the Discussion section:

Line 393: “Low weight gain was consistently associated with increased risk of severe adverse birth outcomes, and in particular with maternal death and perinatal death”

31.	Similarly in Table 4, it is important to note that the adjusted odds ratio for severe adverse birth outcomes is significantly higher in obese women with low weight gain for perinatal death and severe neonatal morbidity, as well as the combined maternal and perinatal adverse outcomes.

**Response:** We agree this is also an important finding. We mentioned this in the Abstract and the Results and Discussion, and we added this finding to the Author Summary:

Line 98: “Weight gain below the recommended range in women who were obese prior to pregnancy may increase the risk of perinatal death or severe neonatal morbidity.”

32.	On page 26, lines 373-377, the authors state that they did not observe any association between excessive weight gain in overweight women in severe adverse maternal and perinatal outcomes. Although these women had a very high rate of excessive weight gain, the authors suggest that the IOM guidelines for optimal weight gain in overweight women may need to be re-evaluated. How would the authors recommend changing the IOM guidelines for overweight women based on their findings?

**Response:** This would need (ideally) a large, population-based study. One option, for instance, would be to focus on changes in the rates of adverse pregnancy outcomes per 1 kg (or other unit) of weight gain among women who were overweight prior to pregnancy and delivered at term. Weight gain values with the largest changes in the rates of adverse pregnancy outcomes (compared to the lowest rates) could be considered as cut-offs for sub-optimal weigh gain, or other alternative criteria could be used based on pre-determined values. Preterm weight gain (per week of gestation) could be then calculated based on the estimated optimal range at term gestation.

33.	In multiple places in the manuscript, for example page 3 line 84 the authors refer to the IOM/AJOG guidelines. AJOG should be ABOG.

**Response:** This was corrected to IOM/ACOG.

**Supplementary addition to Table 1**

|  | **Pre-pregnancy Underweight** | | **Pre-pregnancy Normal BMI** | | **Pre-pregnancy Overweight** | | **Pre-pregnancy**  **Obese** | |
| --- | --- | --- | --- | --- | --- | --- | --- | --- |
|  | **Low weight**  **gain** | **Excess weight gain** | **Low weight gain** | **Excess weight gain** | **Low weight gain** | **Excess weight gain** | **Low weight gain** | **Excess weight gain** |
|  | **N=6,510** | **N=5,967** | **N=73,290** | **N=141,693** | **N=26,284** | **N=11,4632** | **N=37,425** | **N=89,323** |
| **Maternal Age, years:**  15-19 | 0.926 | <0.001 | <0.001 | <0.001 | <0.001 | <0.001 | 0.025 | <0.001 |
| 20-24 | 0.637 | 0.004 | <0.001 | <0.001 | <0.001 | <0.001 | 0.002 | <0.001 |
| 25-29 | 0.098 | 0.002 | <0.001 | 0.100 | 0.045 | <0.001 | 0.037 | 0.096 |
| 30-34 | 0.854 | <0.001 | <0.001 | <0.001 | <0.001 | 0.0157 | 0.195 | <0.001 |
| 35-39 | 0.071 | <0.001 | 0.003 | <0.001 | 0.004 | <0.001 | 0.341 | <0.001 |
| ≥40 | <0.001 | 0.0001 | 0.003 | <0.001 | 0.217 | <0.001 | 0.598 | <0.001 |
| **Race/ethnic:**  Non- Hispanic white | <0.001 | <0.001 | <0.001 | <0.001 | <0.001 | <0.001 | <0.001 | <0.001 |
| African American | <0.001 | 0.095 | <0.001 | 0.004 | <0.001 | <0.001 | <0.001 | <0.001 |
| Native American | 0.587 | <0.001 | <0.001 | <0.001 | 0.012 | 0.076 | 0.005 | 0.99 |
| Hispanic | <0.001 | 0.410 | <0.001 | <0.001 | <0.001 | <0.001 | 0.022 | <0.001 |
| Other | <0.001 | <0.001 | <0.001 | <0.001 | <0.001 | <0.001 | 0.599 | 0.026 |
| Maternal education <high school | <0.001 | 0.444 | <0.001 | <0.001 | <0.001 | <0.001 | <0.001 | <0.001 |
| Smoking | 0.065 | <0.001 | <0.001 | <0.001 | <0.001 | <0.001 | <0.001 | <0.001 |
| Married or common law | 0.4464 | <0.001 | <0.001 | <0.001 | <0.001 | 0.418 | <0.001 | <0.001 |
| **Insurance:** Medicaid | <0.001 | <0.001 | <0.001 | <0.001 | <0.001 | <0.001 | <0.001 | <0.001 |
| Private | <0.001 | <0.001 | <0.001 | <0.001 | <0.001 | <0.001 | <0.001 | <0.001 |
| Self pay | 0.192 | 0.457 | 0.001 | 0.582 | 0.049 | 0.884 | 0.195 | 0.271 |
| Other^b^ | 0.730 | 0.006 | 0.003 | <0.001 | 0.243 | <0.001 | 0.428 | 0.204 |
| **Parity:** Nulliparous | <0.001 | <0.001 | <0.001 | <0.001 | <0.001 | <0.001 | 0.148 | <0.001 |
| 1-3 prior births | <0.001 | <0.001 | <0.001 | <0.001 | <0.001 | <0.001 | 0.048 | <0.001 |
| ≥4 prior births | <0.001 | 0.003 | <0.001 | <0.001 | <0.001 | <0.001 | <0.001 | <0.001 |
| Assisted conception | 0.145 | 0.787 | <0.001 | 0.01228 | 0.002 | 0.032 | 0.164 | 0.900 |
| Pre-pregnancy diabetes mellitus | 1 | 0.191 | <0.001 | <0.001 | 0.071 | 0.960 | 0.029 | 0.964 |
| Chronic hypertension | 0.050 | 0.058 | 0.169 | <0.001 | 0.172 | <0.001 | 0.67 | 0.731 |
| Male fetal sex | 0.001 | 0.058 | <0.001 | <0.001 | 0.352 | <0.001 | <0.001 | <0.001 |
| Congenital anomalies | 0.473 | 0.069 | <0.001 | 0.948 | 0.002 | 0.058 | 0.409 | 0.411 |

Demographic and pregnancy characteristics by pre-pregnancy Body-Mass-Index a and gestational weight gain, singleton births, Washington State, 2004-2013. P-values for differences in these characteristics between optimal weight gain in each pre-pregnancy BMI category.

Abbreviation: BMI, body mass index; O-GWG, optimal gestational weight gain; L-GWG low gestational weight gain; E-GWG, excess gestational weight gain

a Underweight defined as body mass index (BMI, calculated as weight in kilograms divided by height in meters squared) less than 18.5; normal weight as BMI 18.5 through 24.9; overweight as BMI 25.0 through 29.9; obese class I-III as BMI 30.0 or greater.

b Includes other government insurance, student insurance, Indian Health Care, and other programs.
