# Supplementary material for: Association between gestational weight gain and severe adverse birth outcomes in Washington State, US: A population-based retrospective cohort study, 2004–2013
Source: PLoS Med. 2019 Dec 30;16(12):e1003009. doi: 10.1371/journal.pmed.1003009 (PMC6936783; doi:10.1371/journal.pmed.1003009)
Supplement: Attachment — Submitted filename: Response to requests from Editors and production_v2.docx [file pmed.1003009.s012.docx]

------------------------------------------------------------

Dear Editorial Team:

We thank the Editors for provisionally accepting our manuscript. We have made the requested manuscript changes and responded to comments by the editors and the production team below.

**Requests from Editors:**

General comments

E1. Please add reference brackets at the end of each sentence, before the full stop and after a space following the last word.

- **Response:** This has been rectified throughout the manuscript.

E2. Please remove ‘Appendix’ in references to Supplementary Information files

- **Response:** This has also been corrected throughout the manuscript.

E3. Data Availability: The data link provided takes us to a paid for data access site, costing $50 per file, per year. Access to data via a paid service does not comply with our data policy. Please provide a non-payment website where data can be accessed / where authors can apply for access. Please note that a study author cannot be the contact person for the data.

- **Response:** We are not able to legally distribute the data specific to our analyses. The data is available from a third party, and a permission is required.

NOTE: The Department of Health, State of Washington, charges a fee for reviewing the

data request and preparing the requested dataset, including the documentation.

We used this avenue to obtain the data. A fee for data abstraction is common in many

administrative databases (e.g., the Discharge abstract database housed by the Canadian

Institutes for Health Information). We do know about any other data source that would

provide the same information free of charge.

E4. Title – Please include setting and dates of study. We suggest revising the title to “Association between gestational weight gain and severe adverse birth outcomes in Washington, USA: A population-based, retrospective cohort study, 2004-2013”

- **Response:** This has been changed to the suggested title:

“Association between gestational weight gain and severe adverse birth outcomes in Washington State, USA: A population-based retrospective cohort study, 2004-2013”

Abstract

E5. Please begin the Methods and Findings with ‘We conducted’ or similar.

- **Response:** This has been changed to:

**“**We conducted a population-based study of pregnant women with singleton hospital birth in Washington State, USA, between 2004 and 2013”

E6. Lines 42-45: Please consider breaking this sentence up into 3 shorter sentences.

**Response:** We have broken this into three sentences:

“Overall, 722,839 women with information on pre-pregnancy BMI were included. Of these, 3.1% women were underweight, 48.1% had normal pre-pregnancy BMI, 25.8% were overweight, and 23.0% women were obese. Only 31.5% of women achieved optimal gestational weight gain.”

E7. Lines 50-52: Please clarify what is meant by ‘a composite maternal death’ in sentence beginning ‘In addition, low weight gain...’.

- **Response:** This is referring to the occurrence of one or more severe maternal morbidities or maternal death. This has been revised as follows:

“In addition, low weight gain was associated with a composite adverse maternal outcome (death/SMM) in women with normal pre-pregnancy BMI and overweight women…”

Author Summary

E8. Line 71 - please add (IOM) after Institute of Medicine

- **Response:** This has been added

Introduction

E9. Line 122 - please clarify what is meant by ‘delivery hospitalization’

- **Response:** This means woman’s hospital admission for child delivery. his has been to revised to:

`One study reported an overall increased risk of SMM during hospital admission for delivery among women…`

Methods

E10. Line 142 - Please revise to ‘...guideline (S1 Checklist).’

- **Response:** This has been revised

E11. Please rename the prospective analysis plan file as ‘S1 Text’ and include a reference to this your Methods.

- **Response:** This has been revised

Results

E12. Table 2 - please provide definitions for all abbreviated terms used, in the table legend.

- **Response:** These have been included

E13. Tables 3 & 4 - When a p value is given, please specify the statistical test used to determine it.

- **Response:** This has been added under Tables 3 and 4 as:

“2-sided p-values were calculated using multivariable logistic regression Wald Chi-square test**.”**

Discussion

E14. Line 407 - please reformat reference in superscript to PLOS house style (please see below).

- **Response:** This has been revised.

E15. Please add a short section after discussing the strengths/limitations to discuss the implications of the study and next steps for research, clinical practice, and/or public policy

- **Response:** We added the following:

” This study adds to the body of literature that underscores the importance of pre-pregnancy BMI and weight gain during pregnancy to prevent severe adverse pregnancy outcomes. And while these two risk factors are modifiable, we still need effective strategies for women to achieve optimal BMI and weight gain during pregnancy. Pregnancy is an important opportunity to advocate for and support women in behavioural changes towards a healthy lifestyle.”

E16. Please remove the subtitle of ‘Conclusion’

- **Response:** This has been removed

E17. Line 482 - please correct to ‘trimester’

- **Response:** This has been corrected

E18. Figure 1 – Please revise figure to use solid blocks of colour (rather than patterned), and ideally avoid using green and red together.

- **Response:** This has been changed to block and the colours have been changed too.

References

E19. Please make sure references are appropriately capitalised and formatted e.g. ref 11 ‘BMC pregnancy and childbirth’ to ‘BMC Pregnancy and childbirth’

- **Response:** This has been changed

E20. Please use the "Vancouver" style for reference formatting, and see our website for other reference guidelines <https://journals.plos.org/plosmedicine/s/submission-guidelines#loc-references>

- **Response:** These have been reviewed and revised for all references.

E21. Supplementary Files

S1 STROBE Checklist - When completing the checklist, please use section and paragraph numbers, rather than line numbers.

- **Response:** These have been changed as suggested

E22. Tables S3, S4, S6, S7 - please provide definitions for all abbreviated terms used, in the table legend

- **Response:** These have been added for the tables

E23. Tables S4, S5, S8 - When a p value is given, please specify the statistical test used to determine it.

- **Response:** These have been added below the tables

**Response to Production Issues:**

FIGURES and GRAPHICS:

P1. Please remove embedded figures from your manuscript file and ensure the most up to date versions of your figures are uploaded to our submission system as separate TIF or EPS files. Please do not remove the figure legends and captions.

- **Response:** This have been removed

P2. Please ensure that all main figure files are cited in ascending numerical order in the main text of the article.

- **Response:** We have only one figure and this has been cited as suggested in P3 below.

P3. Please ensure that all main figures are referenced in the manuscript as Fig 1, Fig 2, etc. (including capitalization), rather than Figure 1, Figure 2, etc. Please note, however, that the file names themselves must not include the space, i.e. Fig1.tif, Fig2.tif, etc.

- **Response:** This has been revised.

P4. Please ensure that all main figure legends are placed after the paragraph containing their first in-text citation.

- **Response:** This is placed as requested.

P5. Figure 1 has been modified to meet with formatting requirements (specifically, layers were flattened). Please review the revised figure to ensure that it meets with your approval. Please note that if you approve of this revised figure, you do not need to upload it to the system.

- **Response:** We have modified the figure as suggested by the editors (E18); please use the new figure.

TABLES and BOXES:

P5. Please ensure that all main tables in their entirety (including titles and footnotes), are placed after the paragraph containing their first in-text citation.

- **Response:** These have been done for all Tables

P6. Tables 1, 2, 3 and 4 use returns to control text wrapping. This is indicated by a [¶ or ↵] symbol when "Show Paragraph Marks" is turned on in Word. Please either split those cells into separate cells to achieve alignment or remove the returns.

- **Response:** These have been done for all Tables

METADATA:

P7. Please provide a detailed Financial Disclosure statement on the submission form in full sentences, and include URLs, the initials of the receiving author, and specific grant numbers wherever possible.

- **Response:** This has been revised on the submission form as:

“This study was funded by Michael Smith Foundation for Health Research (website: <https://www.msfhr.org/>; Receiving author, SL; Grant no: 16888) and the Canadian Institutes of Health Research (website: <http://www.cihr-irsc.gc.ca/e/193.html> ; Receiving author, SL; Grant no: F17-02161. No, The funders had no role in study design, data collection and analysis, decision to publish, or preparation of the manuscript. )

P8. In accordance with PLOS' data policy, please ensure that your Data Availability Statement in the submission form clearly identifies how readers can access your data. Data cannot be available on request, or only accessible by contacting one of the authors. Note that your Data Availability Statement will be typeset as it is written, so please ensure it is in complete sentences and appears as you would like it to in the published version.

For details on our data policy, please see <http://journals.plos.org/plosmedicine/s/data-availability>

- **Response:** This has been modified, please see response E3.

ARTICLE FILE:

P9. Please indicate the corresponding author in the byline by placing an asterisk (*) after their affiliation number.

- **Response:** This has been added

P10. Please include the corresponding author's email address on the title page of your manuscript, indicated by an asterisk (*). Only an asterisk and the email address itself are required.

- **Response:** This has been revised by removing the address and leaving only the email

P11. Please remove author degree titles from the byline of your article file.

- **Response:** This has been revised

P12. The names for authors U. Vivian Ukah and Sarka Lisonkova do not match across the article file and the submission form. Please ensure that all author names match verbatim, including the use of a middle name/initials and any special characters (accents, umlauts, etc.), as correct spelling and formatting of author names is vital for accurate indexing in PubMed.

- **Response:** These have been corrected

P13. Please ensure that your Ethics statement is available in the Methods section of your manuscript in its entirety.

- **Response:** This has been revised as follows:

“This study was exempted from ethics approval by the Department of Social and Health Services, State of Washington, USA. According to the Washington State Institutional Review Board, a research study can be exempt from the Washington State Institutional Review Board review if “the research does not involve obtaining data about subjects through interaction or intervention with individuals, and does not involve obtaining identifiable private information about subjects from confidential records.” Our study was granted this exemption (DOH Exempt Request E-050515-H).”

SUPPORTING INFORMATION:

P14. Please ensure your supporting information files are individually uploaded to the File Inventory as file type 'Supporting Information' and are not present in the manuscript file.

- **Response:** These have been uploaded as individual files

P15. Please upload your supporting information as individual files, and use the following naming format: S1 Text; S1 Fig; S1 Table, S1 Data, etc. Each file type should start as S1 and increase numerically: S1 Text, S2 Text, etc. Note that all relevant in-text citations and captions will need to be updated accordingly.

- **Response:** These have been uploaded accordingly

P16. Please ensure that all Supporting Information files use the following format verbatim throughout the manuscript (including legends and all in-text citations): S1 Fig, S1 Table, S1 Text, S1 Data, etc. Each file type should start at 1 and increase numerically. Please note, however, that the file names themselves must use an underscore rather than a space, i.e. S1_Fig.tif, S1_Table.xlsx, S1_Text.doc, S1_Data.xlsx, S1_STROBE_Checklist.docx, etc.

- **Response:** These have been revised accordingly

P17. Please ensure that each supporting information file has a legend at the end of the manuscript file, after the Reference list.

- **Response:** These have been revised accordingly

P185. Please note that your submission cannot contain any references to generic "supporting information" files and instead must refer to specific files using our naming convention (S1 Fig, S1 Table, S1 Text, S1 Data, SI PRISMA Checklist, etc.)

- **Response:** These have been revised accordingly

P19. Please replace the page numbers in your STROBE statement with paragraph numbers per section (e.g. "Methods, paragraph 1") since the page numbers of the final published paper may be different from the page numbers in the current manuscript.

- **Response:** These have been revised according (also E21).

P20. Your 'REB proposal' document is currently listed as file type ‘Other’ in the system’s file inventory, meaning it will not publish with the final article. If you would like this document to be published, please change the file type to 'Supporting Information' and ensure it adheres to our naming conventions.

- **Response:** This file can be published as supporting information and has been uploaded as S1_Text.
